# The chicken B-cell line DT40 proteome, beadome and interactomes

**DOI:** 10.1016/j.dib.2014.12.006

**Published:** 2015-01-13

**Authors:** Johanna S. Rees, Kathryn S. Lilley, Antony P. Jackson

**Affiliations:** a Department of Biochemistry, University of Cambridge, Cambridge CB2 1QW, UK; b Cambridge Centre for Proteomics, University of Cambridge, Cambridge CB2 1QR, UK

## Abstract

In developing a new quantitative AP-MS method for exploring interactomes in the chicken B-cell line DT40, we also surveyed the most abundant proteins in this organism and explored the likely contaminants that bind to a variety of affinity resins that would later be confirmed quantitatively [1]. We present the ‘Top 150 abundant DT40 proteins list’, the DT40 beadomes as well as protein interaction lists for the Phosphatidyl inositol 5-phosphate 4-kinase 2β and Fanconi anaemia protein complexes.

**Specifications table**

**Table:** 

Subject area	*Biology*
More specific subject area	*Interactomes of the PIPkinase2 and Fanconi complexes*
Type of data	*Tables and figures*
How data was acquired	*mass spectroscopy using a Thermo hybrid Orbitrap Velos*
Data format	*Analysed using Thermo ProteinCenter and MaxQuant*
Experimental factors	*Pulldowns (all data) with SILAC labelled cell lines(Supplementary*Tables 3 and 4*)*
Experimental features	*Parallel AP-MS using combined SILAC labelled (control) and non labelled (tagged protein)*
Data source location	*Cambridge, UK*
Data accessibility	*Data is with this article. The raw format of the data in Supplementary*Table 2*data has been uploaded to the CRAPome repository for the new release due in Feb 2015 〈http://crapome.org/〉.*

**Value of the data**

• Knowledge of abundant proteins in a Chicken Bcell (DT40) lysate.
• Knowledge of proteins from a Chicken Bcell (DT40) lysate that bind non-specifically to 4 different common affinity resins.
• Genuine and non-specific interacting proteins of Phosphatidyl inositol 5-phosphate 4-kinase 2β.
• Genuine and non-specific interacting proteins of the Fanconi anaemia complex.

## 1 Data

As a prerequisite for applying a new method for studying interactors of Phosphatidyl inositol 5-phosphate 4-kinase 2β and the Fanconi anaemia proteins in DT40, we first wanted to establish the most abundant proteins in a DT40 lysate that could potentially saturate MS data. We refer to this as ‘Top 150 abundant DT40 proteins list’ (Supplementary Table 1) and further look, using the ProteinCenter GO annotation tool, to see which proteins are over and underrepresented (Supplementary Fig. 1).

We then went on to see if these proteins bound non-specifically to an assortment of affinity resins; FLAG, TALON, Calmodulin and IgG and identified hundreds of non-specific interactors for each resin. We compared these lists to identify which proteins bind to multiple resins non-specifically. We refer to this comparative list as the DT40 beadome (Supplementary Table 2) and again performed GO annotation to characterise these proteins (Supplementary Fig. 2).

After establishing the nature of potential non-specific contaminants in AP experiments, we then used a C-terminal tandem 3x FLAG-2x (HIS_6_) tagged Phosphatidyl inositol 5-phosphate 4-kinase 2β protein (PI5P4K2β) (JPR3 cell line) and a C-terminal tandem calmodulin binding protein (CBP) and a-Protein-A tagged FANCC allele (FANCC cell line) to investigate their interacting partners. We coupled this with SILAC labelling to make analysis quantitative (qAP-MS), thus enabling us to discriminate genuine from non-specific interactors. It is already known that the nuclear membrane protein PI5P4K2β forms homodimers and also heterodimerises with PI5P4K2α with no other proteins interacting, not surprisingly, as its substrates are lipids. The MS identified interactors and isotopic ratios are presented as Supplementary Table 3A and B for FLAG and TALON affinity purifications respectively.

The Fanconi complex however is less well understood and interactors are transient and of low abundance upon stalling of replication forks. We therefore wished to investigate this complex more thoroughly and identified some new interactors of which a couple were independently confirmed (genetically) by another group. The resulting partners and their quantitative analysis are presented in Supplementary Table 4.

## 2 Experimental design, materials and methods

In initial studies to identify abundant DT40 proteins, three replicates of 10^6^ total cells, grown in RPMI media with l-glutamine supplemented with 10% FBS and 1% chicken serum (CS) (all GIBCO) and maintained at 37 °C at 5% CO_2_, as described previously [2], were lysed in 10 ml lysis buffer, comprising 1× PBS (pH7.4) 1% Triton X-100, 1 mM PMSF and 1.5× EDTA free protease inhibitor cocktail (Roche), on ice and the cleared supernatants separated by reducing SDS-PAGE and stained. All bands were excised and the sample prepared and analysed by MS as described below.

For DT40 beadomes and SILAC-iPAC experiments all cell lines were grown in parallel to 10^8^ total cells. Cells were lysed in 10 ml lysis buffer on ice and the cleared supernatants quantified, then added to 100 μl prewashed resins. For SILAC-iPAC experiments quantified lysates were mixed 1:1 (typically 5 mg) then added to 100 μl prewashed resins. JPR3 and wt DT40 cell extracts were purified using EZview ANTI-FLAG M2 Affinity Gel (Sigma) and TALON Metal Affinity Resin (Clontech). FANCC and wild-type DT40 cell extracts were purified with Calmodulin Sepharose 4B and IgG Sepharose 6 (both GE Healthcare). Native protein complexes were allowed to bind for 1 h then non-bound proteins were removed by centrifugation at 2000*g*, the resins were washed 3 times for 15 min in 1 ml lysis buffer and the bound proteins were eluted in 100 μl of elution buffer. Elution buffers were lysis buffer pH 7.4 containing either 100 μg/ml 3× FLAG peptide for FLAG resin, 150 mM imidazole for TALON resins, or 200 mM EDTA for calmodulin resins. 100 mM glycine pH2.5 was used for IgG resins.

### 2.1 Sample preparation for MS/MS

Eluates were acetone precipitated overnight at −80 °C, resuspended in LDS sample buffer and resolved for ~2 cm on 10% reducing SDS-PAGE gels (Invitrogen). Analysis gels were Coomassie stained and four equal sized portions of the stained area were excised, washed, reduced in 2 mM DTT for 1 h at RT, alkylated in 10 mM iodoacetamide for 30 min at RT, and in-gel digested with 2 μg sequencing-grade porcine trypsin (Promega) overnight at 37 °C. Digests were concentrated using a speedvac and resuspended in 20 μl 0.1% formic acid.

### 2.2 Mass spectrometry

All LC–MS/MS experiments were performed using a nanoAcquity UPLC (Waters Corp., Milford, MA) system coupled to an LTQ Orbitrap Velos hybrid ion trap mass spectrometer (Thermo Scientific, Waltham, MA). Separation of peptides was performed by reverse-phase chromatography using a Waters reverse-phase nano column (BEH C18, 75 μm i.d. ×250 mm, 1.7 μm particle size) at flow rate of 300 nL/min. 10 μl peptide sample solution was initially loaded onto a pre-column (Waters UPLC Trap Symmetry C18, 180 μm i.d. ×20 mm, 5 μm particle size) from the nanoAcquity sample manager with 0.1% formic acid for 3 min at a flow rate of 10 μl/min. After this period, the column valve was switched to allow the elution of peptides from the pre-column onto the analytical column. Solvent A was water+0.1% formic acid and solvent B was acetonitrile+0.1% formic acid. The linear gradient employed was 5–35% B in 60 min. Washes with 0.1% formic acid were performed in between each biological sample type to minimise carryover. The Velos was operated in data dependent mode with a dynamic exclusion of 0.3 Da *m*/*z*.

The LC eluant was sprayed into the mass spectrometer by means of a nanospray source (Thermo). All *m/z* values of eluting ions were measured in the Orbitrap Velos mass analyzer, set at a resolution of 30,000. Data dependent scans (Top 10) were employed to automatically isolate and generate fragment ions by collision-induced dissociation in the linear ion trap, resulting in the generation of MS/MS spectra. Ions with charge states of 2+ and above were selected for fragmentation.

### 2.3 Protein identification

Post-run, the data was processed using Protein Discoverer (version 1.2., ThermoFisher). Briefly, all MS/MS data were converted to.mgf files and these files were then submitted to the Mascot search algorithm (v 2.3, Matrix Science, London UK) and searched against the UniprotKB *Gallus gallus* database (31,529 protein entries, 2012), using a fixed modification of carbamidomethyl (C) and a variable modification of oxidation (M), allowing 2 missed cleavages, a peptide mass tolerance of 25 ppm and a fragment mass tolerance of 0.8 Da. Significance threshold was set to *P*>0.01and ion score cut off was set to 20. FDR was calculated using the reverse *Gallus* database and only proteins with an FDR of <5% were considered. In addition Mascot Percolator was used for scoring and to compare protein lists. For quantitation we used MaxQuant v 1.3 [3]. Default parameters were used with FDR of 0.01%, and a minimum of two unique and razor peptides were selected for quantifying proteins. We used the MaxQuant generated normalised data for quantitative comparison across replicates and reciprocal labelling experiments and used Microsoft excel to plot the Log_2_ ratio distributions. We set a significance threshold of a ratio >±1 SD of the median of the entire normalised data set. Data processing and filtering is outlined in Supplementary Fig. 3. Venn diagrams were produced using 〈http://bioinfogp.cnb.csic.es/tools/venny/index.html〉 [4].

### 2.4 DT40 abundant proteins (Supplementary Table 1 and Fig. 1)

Data (Supplementary Table 1) was further processed using ProteinCenter Version 3.13.2003 (Thermo) using Mascot xml outputs processed. Data shows the top 150 abundant proteins from 3 replicate DT40 lysate analyses, ranked by emPAI score. From GO annotation (Supplementary Fig. 1) we clearly see a bias and overrepresentation of ribosomes, viral proteins, vesicular and mitochondrial proteins. The under-represented proteins included membrane and extracellular matrix proteins. This analysis is important in understanding the likely abundant proteins that may mask genuine low abundance proteins from AP-MS experiments.

### 2.5 The DT40 beadome: proteins that bind non-specifically to FLAG, TALON, calmodulin and IgG resins (Supplementary Table 2 and Fig. 2)

Data (Supplementary Table 2) was further processed using ProteinCenter as described previously. Data shows the top 150 DT40 proteins identified that bind non-specifically to the resins, FLAG, TALON, Calmodulin and IgG. The DT40 beadomes identified a total of 367 proteins (1.16% of the *Gallus* proteome) of which 150 (41%) bound to two or more different affinity resins (Supplementary Table 2). These proteins were predominantly cytoplasmic and ribosomal as classified by Gene Ontology and Kegg pathway annotations (Supplementary Fig. 2). Almost half (45%) of these proteins were present in the top 150 abundant DT40 protein list highlighting the fact that abundant proteins can non-specifically bind any affinity resin and mask genuine interactors.

These initial studies provide data on the nature of potential contaminants, and further highlight the problem of discriminating specific interactors from non-specific proteins in any type of affinity purification of native complexes.

### 2.6 The PI5P4K2β interactomes (Supplementary Table 3A and B)

A total of 16 replicate qAP-MS experiments were performed to see if we could find further interactors of the well-established enzyme PI5P4K2β. Data (Supplementary Table 3) shows PI5P4K2β associated statistically significant proteins from replicate datasets that were pulled down with FLAG (A) and TALON resins (B) with proteins in common to both highlighted in green. Here only 18 proteins of >500 replicate identifications were in common to both pulldowns and of these only the PI5P4K2β and PI5P4K2α had reproducible significant ratios demonstrating their dominance amongst a plethora of co-eluting proteins. We found no other experimental evidence or validation (using STRING) of these other interacting partners and none seemed biologically meaningful thus we classified these proteins as non-specific. Full details and analysis can be found in the accompanied manuscript in press [1].

### 2.7 The Fanconi interactomes (Supplementary Table 4A–C)

A total of 8 replicate qAP-MS experiments were performed to see if we could find further interactors of FANCC. Data (Supplementary Table 4) shows FANCC associated proteins that had at least one statistically significant protein from replicate datasets that were pulled down with both calmodulin and IgG pulldowns (A), calmodulin resins (B) or IgG resins only (C). Here we found a much higher overlap of interactors pulled down with the two different resins. Many more were statistically significant and some were independently validated [5] or confirmed using STRING mapping. Full details and analysis can be found in the accompanied manuscript in press [1].

## Footnotes

Appendix A Supplementary data associated with this article can be found in the online version at doi:10.1016/j.dib.2014.12.006.
