# Supplementary material for: The chicken B-cell line DT40 proteome, beadome and interactomes
Source: Data Brief. 2015 Jan 13;3:29–33. doi: 10.1016/j.dib.2014.12.006 (PMC4509924; doi:10.1016/j.dib.2014.12.006)

**Figure 1.** GO annotation and proteins over- and under-represented in DT40 lysate.

***A***) Pie chart showing the GO cellular components classification of DT40

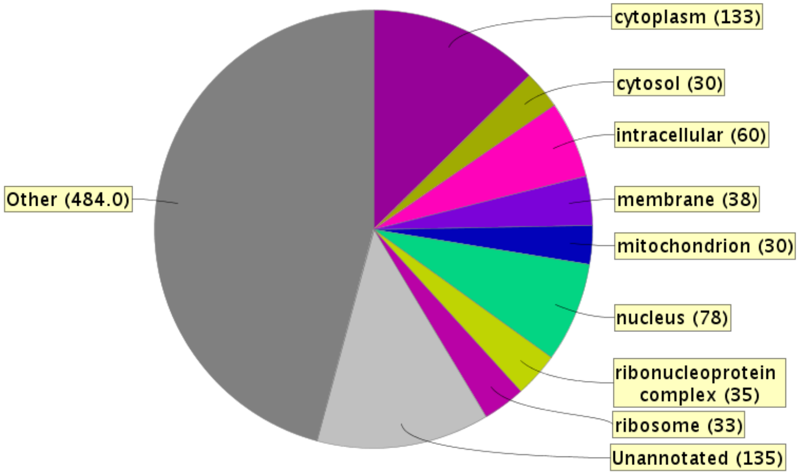


***B***) Proteins under-represented in DT40 compared to the *Gallus gallus* SwissProt proteome

| Description | Count [%] | Ref. Count [%] | Count | Ref. Count | Raw p-value | FDR p-value |
|---|---|---|---|---|---|---|
| cellular_component | 73.38 | 89.85 | 306 | 2143 | 7.29E-28 | 3.99E-24 |
| intrinsic to membrane | 6.95 | 22.39 | 29 | 534 | 4.20E-20 | 3.28E-17 |
| integral to membrane | 6.95 | 21.3 | 29 | 508 | 4.17E-18 | 1.14E-15 |
| extracellular region | 2.16 | 12.87 | 9 | 307 | 5.62E-17 | 1.28E-14 |
| membrane | 17.75 | 33.88 | 74 | 808 | 9.93E-16 | 2.05E-13 |
| membrane part | 12.47 | 25.83 | 52 | 616 | 2.70E-13 | 4.62E-11 |
| plasma membrane | 5.04 | 14.68 | 21 | 350 | 1.18E-11 | 1.90E-09 |
| cell periphery | 6.24 | 15.51 | 26 | 370 | 2.92E-10 | 4.31E-08 |
| nucleus | 21.1 | 33 | 88 | 787 | 3.29E-09 | 4.61E-07 |
| intracellular membrane-bounded organelle | 34.53 | 46.96 | 144 | 1120 | 1.14E-08 | 1.52E-06 |
| membrane-bounded organelle | 34.53 | 46.96 | 144 | 1120 | 1.14E-08 | 1.52E-06 |
| extracellular matrix | 0 | 3.35 | 0 | 80 | 1.58E-07 | 1.96E-05 |
| proteinaceous extracellular matrix | 0 | 3.1 | 0 | 74 | 5.21E-07 | 5.82E-05 |
| extracellular region part | 1.68 | 6.16 | 7 | 147 | 1.98E-06 | 2.04E-04 |
| chromosomal part | 0.96 | 4.07 | 4 | 97 | 5.37E-05 | 3.65E-03 |
| chromosome | 1.44 | 4.86 | 6 | 116 | 5.40E-05 | 3.65E-03 |
| cell junction | 0.72 | 3.4 | 3 | 81 | 1.41E-04 | 8.45E-03 |
| cell | 66.19 | 73.5 | 276 | 1753 | 1.60E-04 | 9.14E-03 |
| cell part | 66.19 | 73.5 | 276 | 1753 | 1.60E-04 | 9.14E-03 |
| extracellular matrix part | 0 | 1.59 | 0 | 38 | 6.32E-04 | 2.91E-02 |
| chromatin | 0.24 | 2.1 | 1 | 50 | 7.13E-04 | 3.20E-02 |
| synapse | 0.24 | 2.1 | 1 | 50 | 7.13E-04 | 3.20E-02 |
| plasma membrane part | 2.16 | 5.12 | 9 | 122 | 8.39E-04 | 3.70E-02 |

***C***) Proteins over-represented in DT40 compared to the *Gallus gallus* SwissProt proteome

| Description | Count [%] | Ref. Coun | Count | Ref. Coun | Raw p-value | FDR p-valu |
|---|---|---|---|---|---|---|
| ribosome | 7.91 | 1.34 | 33 | 32 | <4.90E-324 | <4.90E-324 |
| virion | 3.12 | 0.13 | 13 | 3 | <4.90E-324 | <4.90E-324 |
| virion part | 3.12 | 0.13 | 13 | 3 | <4.90E-324 | <4.90E-324 |
| viral capsid | 2.88 | 0.04 | 12 | 1 | <4.90E-324 | <4.90E-324 |
| cytosolic large ribosomal subunit | 1.68 | 0.25 | 7 | 6 | <4.90E-324 | <4.90E-324 |
| proteasome core complex | 1.68 | 0.13 | 7 | 3 | <4.90E-324 | <4.90E-324 |
| clathrin coat | 1.2 | 0.13 | 5 | 3 | <4.90E-324 | <4.90E-324 |
| proteasome core complex, alpha-subunit complex | 0.96 | 0.08 | 4 | 2 | <4.90E-324 | <4.90E-324 |
| proton-transporting ATP synthase complex, catalytic core F(1) | 0.96 | 0.04 | 4 | 1 | <4.90E-324 | <4.90E-324 |
| chaperonin-containing T-complex | 0.72 | 0 | 3 | 0 | <4.90E-324 | <4.90E-324 |
| clathrin coat of coated pit | 0.48 | 0.04 | 2 | 1 | <4.90E-324 | <4.90E-324 |
| clathrin coat of trans-Golgi network vesicle | 0.48 | 0 | 2 | 0 | <4.90E-324 | <4.90E-324 |
| clathrin vesicle coat | 0.48 | 0 | 2 | 0 | <4.90E-324 | <4.90E-324 |
| septin complex | 0.48 | 0.04 | 2 | 1 | <4.90E-324 | <4.90E-324 |
| trans-Golgi network transport vesicle | 0.48 | 0 | 2 | 0 | <4.90E-324 | <4.90E-324 |
| trans-Golgi network transport vesicle membrane | 0.48 | 0 | 2 | 0 | <4.90E-324 | <4.90E-324 |
| fatty acid beta-oxidation multienzyme complex | 0.24 | 0 | 1 | 0 | <4.90E-324 | <4.90E-324 |
| mitochondrial fatty acid beta-oxidation multienzyme complex | 0.24 | 0 | 1 | 0 | <4.90E-324 | <4.90E-324 |
| mitochondrial proton-transporting ATP synthase complex, catalytic core F(1) | 0.24 | 0 | 1 | 0 | <4.90E-324 | <4.90E-324 |
| perikaryon | 0.24 | 0 | 1 | 0 | <4.90E-324 | <4.90E-324 |
| ribonucleoside-diphosphate reductase complex | 0.24 | 0 | 1 | 0 | <4.90E-324 | <4.90E-324 |
| ribosomal subunit | 0.24 | 0 | 1 | 0 | <4.90E-324 | <4.90E-324 |
| signal recognition particle receptor complex | 0.24 | 0 | 1 | 0 | <4.90E-324 | <4.90E-324 |
| ribonucleoprotein complex | 10.07 | 2.98 | 42 | 71 | 1.27E-15 | 8.47E-15 |
| ribosomal subunit | 3.84 | 0.67 | 16 | 16 | 5.99E-13 | 3.87E-12 |
| cytosolic part | 4.32 | 1.05 | 18 | 25 | 2.44E-09 | 1.52E-08 |
| cytosol | 10.55 | 4.82 | 44 | 115 | 4.54E-08 | 2.76E-07 |
| large ribosomal subunit | 2.16 | 0.38 | 9 | 9 | 1.42E-07 | 8.34E-07 |
| proteasome complex | 2.16 | 0.38 | 9 | 9 | 1.42E-07 | 8.34E-07 |
| cytosolic ribosome | 2.64 | 0.55 | 11 | 13 | 2.32E-07 | 1.34E-06 |
| macromolecular complex | 29.5 | 20.46 | 123 | 488 | 7.79E-07 | 4.40E-06 |
| cytoplasmic part | 35.01 | 25.49 | 146 | 608 | 1.14E-06 | 6.41E-06 |
| cytoplasm | 52.28 | 42.22 | 218 | 1007 | 3.41E-06 | 1.89E-05 |
| small ribosomal subunit | 1.68 | 0.29 | 7 | 7 | 4.79E-06 | 2.61E-05 |
| coated membrane | 1.68 | 0.34 | 7 | 8 | 3.25E-05 | 1.71E-04 |
| membrane coat | 1.68 | 0.34 | 7 | 8 | 3.25E-05 | 1.71E-04 |
| Golgi-associated vesicle | 1.2 | 0.21 | 5 | 5 | 1.60E-04 | 8.00E-04 |
| Golgi-associated vesicle membrane | 1.2 | 0.21 | 5 | 5 | 1.60E-04 | 8.00E-04 |
| proton-transporting ATP synthase complex | 0.96 | 0.17 | 4 | 4 | 9.23E-04 | 3.99E-03 |
| vesicle coat | 0.96 | 0.17 | 4 | 4 | 9.23E-04 | 3.99E-03 |
| intracellular non-membrane-bounded organelle | 21.58 | 16.44 | 90 | 392 | 1.47E-03 | 6.21E-03 |
| non-membrane-bounded organelle | 21.58 | 16.44 | 90 | 392 | 1.47E-03 | 6.21E-03 |
| proton-transporting two-sector ATPase complex | 1.68 | 0.5 | 7 | 12 | 1.68E-03 | 7.07E-03 |
| coated vesicle membrane | 1.44 | 0.42 | 6 | 10 | 3.08E-03 | 1.28E-02 |
| coated vesicle | 1.68 | 0.55 | 7 | 13 | 3.10E-03 | 1.29E-02 |
| proton-transporting two-sector ATPase complex, catalytic domain | 0.96 | 0.21 | 4 | 5 | 3.98E-03 | 1.61E-02 |
| AP-type membrane coat adaptor complex | 0.72 | 0.13 | 3 | 3 | 5.31E-03 | 2.06E-02 |
| clathrin adaptor complex | 0.72 | 0.13 | 3 | 3 | 5.31E-03 | 2.06E-02 |
| COPI coated vesicle membrane | 0.72 | 0.13 | 3 | 3 | 5.31E-03 | 2.06E-02 |
| COPI-coated vesicle | 0.72 | 0.13 | 3 | 3 | 5.31E-03 | 2.06E-02 |
| phosphopyruvate hydratase complex | 0.72 | 0.13 | 3 | 3 | 5.31E-03 | 2.06E-02 |
| cytoskeletal part | 9.35 | 6.37 | 39 | 152 | 5.70E-03 | 2.18E-02 |

**Figure 2.** Non-specific binding proteins associated with ≥2 of 4 affinity resins: FLAG, Talon, Calmodulin and IgG sepharose. *A*) Pie chart showing GO_Cellular component annotation. *B*) GO_Molecular function annotation and *C*) Kegg pathway analysis. 'Other' represents proteins other categories with single protein entries. Data analysed using ProteinCenter. *D*) Overlap of proteins identified as top 150 abundant and the top 150 beadome.

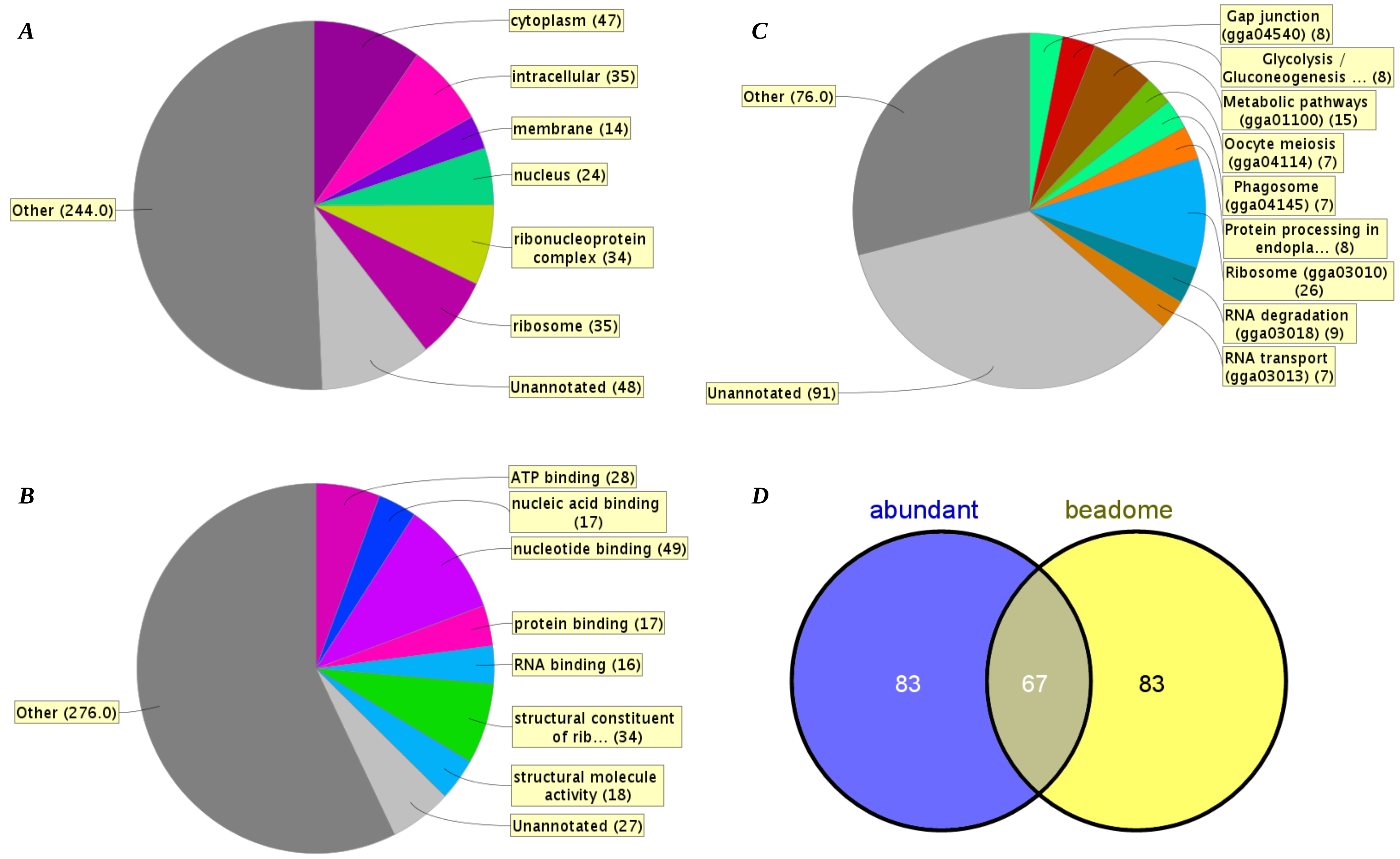

**Figure 3.** Mass Spectrometry data workflow and protein acceptance criteria for the quantitative interactomes.

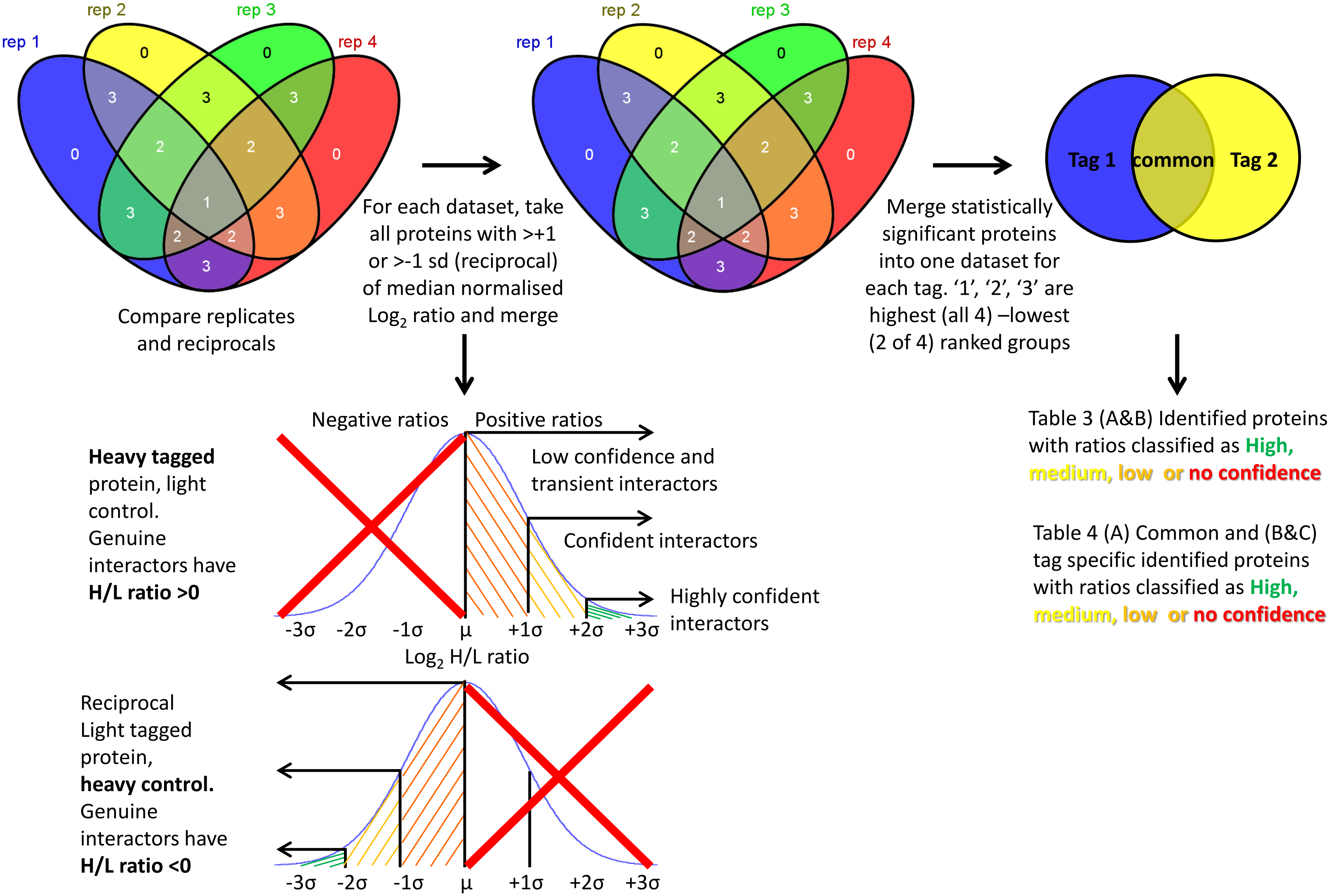

Supplement: Supplementary file 1 — Supplementary data [file mmc1.zip › Figures 1-3.pdf]
