# Supplementary material for: The chicken B-cell line DT40 proteome, beadome and interactomes
Source: Data Brief. 2015 Jan 13;3:29–33. doi: 10.1016/j.dib.2014.12.006 (PMC4509924; doi:10.1016/j.dib.2014.12.006)
Supplement: Supplementary file 1 — Supplementary data [file mmc1.zip › Table 1.pdf]

Table 1. Top 150 abundant DT40 proteins from 3 LC-MS/MS runs ranked by calculated emPAI score.

UNIPROTKB	GENE	MASCOT SCORE AVERAGE	EMPAI AVERAGE	EMPAI CALC AVERAGE	UNIQUE PEPTIDES	UNIQUE PEPTIDE SEQS	% SEQUENCE COVERAGE	DESCRIPTION
O42283	HSPE1	263	2.87	9.00	10	10	85.29	Heat shock protein 10
G1K342	TUBB2C	2613	7.13	7.91	33	21	66.29	Uncharacterized protein
P09244	TUBB	2992	8.32	7.91	33	22	72.52	Tubulin beta-7 chain
Q5ZLN1	PGAM1	1267	4.66	7.25	14	12	71.26	Phosphoglycerate mutase 1
Q6EE57	RPS8	408	1.69	6.94	12	12	60.32	40S ribosomal protein S8
P53478	ACTG1	2139	10.18	6.74	29	22	66.22	Actin, cytoplasmic type 5
Q90705	EEF2	2925	3.21	6.74	56	50	66.55	Elongation factor 2
P51913	ENO1	7909	8.12	6.59	29	26	63.82	Alpha-enolase
B4ZAD9	EEF2	530	2.73	6.20	17	14	72.34	Eukaryotic translation elongation factor 2
P11501	HSP90AA1	3043	4.35	5.81	51	45	57.01	Heat shock protein HSP 90-alpha
P00356	GAPDH	1741	5.37	5.49	25	16	59.76	glyceraldehyde-3-phosphate dehydrogenase
Q9IAY5	SDOS	609	2.06	5.49	13	13	45.00	protein syndesmos precursor
Q5ZLQ6	YWHAB	989	2.86	5.31	15	14	59.84	14-3-3 protein beta/alpha
P00340	LDHA	2790	8.44	4.93	29	21	78.61	L-lactate dehydrogenase A chain
Q5ZL72	HSPD1	2410	4.36	4.74	33	27	57.77	60 kDa heat shock protein, mitochondrial
O57535	NME2	531	9.02	4.62	11	9	74.51	Nucleoside diphosphate kinase
P09203	TUB1C	2232	4.92	4.62	26	17	54.38	Tubulin beta-1 chain
Q6EE60	RPL18	281	1.68	4.62	7	7	46.39	60S ribosomal protein L18
Q98TF8	RPL22	271	0.51	4.62	4	4	26.56	60S ribosomal protein L22
P00337	LDHB	946	2.65	4.46	17	17	61.56	L-lactate dehydrogenase B chain
P42558	RAN	296	1.45	4.34	8	8	42.59	GTP-binding nuclear protein Ran
A3RL83	ANP32B	292	0.97	4.01	9	9	30.38	Lectin-associated matrix protein
E1C2A1	ANP32A	274	0.98	4.01	9	9	36.30	Acidic leucine-rich nuclear phosphoprotein 32 family member
O73885	HSPA8	2295	3.24	3.98	33	30	52.79	Heat shock cognate 71 kDa protein
Q5ZKC9	YWHAZ	1100	2.86	3.87	15	14	53.06	14-3-3 protein zeta
P63247	GNB2L1	690	1.44	3.83	15	15	47.95	Guanine nucleotide-binding protein subunit beta-2-like 1
Q5ZMN3	PHB2	438	0.94	3.81	19	18	57.81	Prohibitin-2
P00940	TPI1	465	2.22	3.64	11	11	64.11	Triosephosphate isomerase
P04210	L1CHV	266	1.27	3.64	4	4	54.72	Ig lambda chain V-1 region
Q5ZKK8	RPL19	26	0.14	3.64	8	8	35.20	Ribosomal protein L19
Q5ZLC5	ATP5B	1662	2.67	3.64	28	22	59.29	ATP synthase subunit beta, mitochondrial
Q5ZMD1	YWHAQ	537	1.74	3.64	12	11	44.49	14-3-3 protein theta
Q5ZMT0	YWHAE	837	3.05	3.64	15	14	48.63	14-3-3 protein epsilon
Q90ZG0	FKBP1A	42	0.66	3.64	2	2	25.00	Peptidyl-prolyl cis-trans isomerase
Q5ZJU3	ASNS	885	1.10	3.52	20	19	40.29	Asparagine synthetase [glutamine-hydrolyzing]
Q8JFP1	EIF4A2	857	1.99	3.49	19	19	47.67	Eukaryotic initiation factor 4A-II
F2Z4K7	RPS3A	143	1.08	3.44	15	14	50.76	Uncharacterized protein
Q90835	EEF1A1	799	2.12	3.44	16	15	38.96	Elongation factor 1-alpha 1
P47836	RPS4X	373	3.39	3.28	17	16	53.23	40S ribosomal protein S4
Q5ZJC1	RPS3	358	1.26	3.28	13	13	54.51	Putative uncharacterized protein
P79781	RPS27A	235	0.97	3.22	7	6	36.54	Ubiquitin-40S ribosomal protein S27a
Q90694	CDC42	80	1.37	3.22	5	5	32.46	Cell division control protein 42 homolog
F1N9J7	TUBA3E	1164	1.74	3.16	17	15	41.78	PREDICTED: tubulin alpha-1C chain
Q9I9V6	GAG	193	0.47	3.12	8	7	26.42	Gag polyprotein
P00548	PKM2	2455	3.64	3.08	30	24	56.79	Pyruvate kinase muscle isozyme
P47826	RPLP0	998	2.02	3.08	11	11	49.68	60S acidic ribosomal protein P0
P68034	ACTC1	927	3.18	3.08	19	15	35.81	Actin, alpha cardiac muscle 1
F1P1A5	TKT	735	1.20	2.98	22	21	36.36	PREDICTED: transketolase

P02552	TUBA1C	806	2.47	2.98	6	6	40.56	Tubulin alpha-1 chain
Q5ZJ56	RPL7	66	0.93	2.98	11	9	38.62	60S ribosomal protein L7
Q9I9V5	GAG	258	2.08	2.98	8	7	33.89	Gag polyprotein
Q9I9V7	GAG	215	1.39	2.98	8	7	33.89	Gag polyprotein
D5M8S3	PHB	350	0.88	2.87	11	11	52.94	Prohibitin transcript variant 2
P15771	NCL	1184	1.15	2.86	28	25	35.73	nucleolin/C23
D0EKR3	PPIA	528	5.56	2.83	13	9	44.24	Peptidyl-prolyl cis-trans isomerase
F1NGS7	ARF4	94	0.83	2.83	7	7	40.56	Uncharacterized protein
P09653	TUBB6	938	1.14	2.79	19	12	33.41	Tubulin beta-5 chain
Q5F424	CCT2	1070	1.57	2.76	26	22	52.52	Uncharacterized protein
O93466	RAC3	104	0.33	2.73	6	6	26.04	GTPase cRac1B
Q6EE31	CCT8	710	1.91	2.73	28	24	46.53	T-complex protein 1 subunit theta
Q9W7I5	RBBP4	157	0.31	2.73	8	8	19.29	Histone-binding protein RBBP4
P38024	PAICS	747	0.95	2.67	18	15	45.77	Multifunctional protein ADE2
Q5ZJK8	CCT7	1323	1.45	2.65	23	20	45.03	T-complex protein 1 subunit eta
P51903	PGK1	774	2.57	2.63	19	17	47.00	Phosphoglycerate kinase
P20763		134	0.50	2.59	5	5	40.37	Ig lambda chain C region
Q6ITC7	RPS13	44	0.43	2.59	5	5	39.74	40S ribosomal protein S13
Q6SVA6	RPSA	269	0.62	2.59	7	7	33.77	DMRT1 isoform e
P09652	TUBB3	1518	2.10	2.55	20	12	32.96	Tubulin beta-4 chain
Q6JAY6	GAG	197	1.55	2.51	7	7	26.27	Gag polyprotein
Q9PTD6	RPS6	105	0.56	2.51	8	7	35.22	40S ribosomal protein S6
Q9I9V4	GAG	379	1.54	2.46	12	9	30.83	Gag polyprotein
Q90679	THYN1	103	0.75	2.38	9	9	43.80	Thymocyte nuclear protein 1
Q9DEA3	PCNA	194	1.37	2.38	12	10	45.80	Proliferating cell nuclear antigen
Q8UVX3	ATP5A1	1216	1.22	2.27	17	17	38.34	ATP synthase subunit alpha
Q5ZL53	PABPC1	422	0.80	2.27	21	20	35.32	Putative uncharacterized protein
Q04619	HSP90AB1	966	0.92	2.26	27	26	33.38	Heat shock cognate protein HSP 90-beta
F1NG87	TTC28	24		2.16	3	3	26.09	similar to OTTHUMP00000028696
F1P3F1	AHCY	456	0.70	2.16	14	13	39.26	adenosylhomocysteinase
H9KZC8	PA2G4	191	1.09	2.16	13	13	42.27	similar to proliferation-associated protein 1, partial
O42388	UBA52	129	0.51	2.16	5	4	25.78	Ubiquitin-ribosomal protein fusion protein
P16039	NPM1	224	0.62	2.16	11	11	34.01	Nucleophosmin
P18359	DSTN	354	2.72	2.16	7	6	44.85	Destrin
P18660	RPLP1	63	0.30	2.16	1	1	14.04	60S acidic ribosomal protein P1
P53449	ALDOC	486	1.86	2.16	4	3	35.04	Fructose-bisphosphate aldolase C
Q02960	MIF	107	1.05	2.16	5	4	34.78	Macrophage migration inhibitory factor
Q5F3R9	SET	136	0.48	2.16	5	5	28.16	Putative uncharacterized protein
Q5ZHW8	RPS14	200	0.45	2.16	4	4	28.48	Uncharacterized protein
Q5ZL59	UBE2D3	59	0.20	2.16	2	2	23.81	Uncharacterized protein
Q6EE59	RPL4	184	0.64	2.16	8	8	35.36	Ribosomal protein L4
Q6EE62	RPL10A	66	0.35	2.16	5	5	28.80	Ribosomal protein
Q6JAX8	GAG	163	1.26	2.16	7	7	26.27	Gag polyprotein
P09102	P4HB, PDI, PDIA1	395	0.39	2.05	18	18	38.21	protein disulfide isomerase
Q5F411	CCT5	678	0.53	2.05	19	18	32.72	Uncharacterized protein
F6R1X6	SSB	205	0.41	2.03	18	18	43.56	Sjogren syndrome antigen B (autoantigen La) isoform 1
Q5ZIQ3	HNRPK	203	0.22	2.02	11	11	35.83	Heterogeneous nuclear ribonucleoprotein K
F1NGA2	ATP5A1W	843	1.12	2.01	11	11	32.40	ATP synthase alpha subunit
P08110	HSP90B1, TRA1	449	0.32	1.99	22	22	26.67	Endoplasmin
Q5F470	RAB8A	33	0.30	1.93	7	7	40.10	Ras-related protein Rab-8A
Q9W744	DDX5	270	0.27	1.93	17	16	27.39	DEAD-box RNA helicase
Q03853	SE21Q1b	649	0.98	1.91	19	15	28.67	Gag protein
Q8UWG7	RPL6	84	0.32	1.89	7	7	22.82	60S ribosomal protein L6
Q9I9V2	GAG	365	1.18	1.89	10	7	26.94	Gag polyprotein
Q5F4A4	IMPDH2	438	0.67	1.87	17	17	37.55	Inosine-5'-monophosphate dehydrogenase
H9L011	LOC426023	126	0.57	1.85	5	5	32.12	nuclear poly(C)-binding protein, splicevariant E

F1NXW3	RPS15A	127	1.78	1.85	8	7	42.31	40S ribosomal protein S15a isoform 1
P41125	RPL13	126	0.67	1.85	7	7	28.44	60S ribosomal protein L13
Q5ZKJ3	DUT	134	0.68	1.85	5	5	30.72	Uncharacterized protein
Q6JAY3	GAG	220	1.55	1.85	8	7	33.86	Gag polyprotein
P09207	TUBB1	628	0.76	1.82	15	10	24.44	Tubulin beta-6 chain
Q03852	RAV-0	703	1.25	1.81	19	15	29.10	Gag protein
P51417	RPL15	74	0.90	1.78	6	6	42.42	60S ribosomal protein L15
Q5F4A3	ANP32E	113	0.54	1.78	4	4	19.92	Acidic leucine-rich nuclear phosphoprotein 32 family member
Q5ZJ54	CCT6A	852	0.64	1.78	14	13	31.51	T-complex protein 1 subunit zeta
Q5ZME2	MDH1	91	0.19	1.78	10	9	30.54	Malate dehydrogenase, cytoplasmic
Q9DDH7	ATP5A1	217	1.13	1.78	4	4	39.09	ATP synthase alpha subunit
O93382	GDI2	746	0.99	1.75	15	13	39.06	Rab-GDP dissociation inhibitor
H9KZF8	ATP5C1	50	0.47	1.68	6	6	22.87	similar to ATP synthase, H+ transporting, mitochondrial F1 co
P00508	GOT2	494	0.60	1.68	16	16	37.59	Aspartate aminotransferase, mitochondrial
Q6EE30	EEF1G	401	0.89	1.68	11	11	24.77	Eukaryotic translation elongation factor 1
Q5ZMU3	GPI	712	0.85	1.65	18	16	28.93	Glucose-6-phosphate isomerase
Q5ZIX2	ETFA	191	0.29	1.64	8	8	30.92	Putative uncharacterized protein
P49702	ARF5	103	0.58	1.61	5	5	31.11	ADP-ribosylation factor 5
Q5ZJX9	PSMA5	29	0.42	1.61	5	5	23.24	Proteasome subunit alpha type
Q5ZMT1	ARHGDIA	152	0.71	1.61	5	4	38.24	Putative uncharacterized protein
Q90WA6	HSP90B1	449	0.33	1.60	20	20	24.03	Heat shock protein 108
E1C4M0	RPS2	290	1.64	1.51	10	10	29.53	40S ribosomal protein S2
H9L366	IDH2	45	0.42	1.51	2	2	50.67	hypothetical protein LOC431056
D0VX32	UQCRQ	40	0.35	1.51	2	2	28.24	Mitochondrial ubiquinol-cytochrome c reductase ubiquinone-
P09643	TUBA4A	2112	1.60	1.51	9	7	25.16	Tubulin alpha-4 chain
Q5ZI56	RPL11	199	0.52	1.51	5	5	26.53	Putative uncharacterized protein
F1NSQ1	AIMP2	82	0.11	1.42	6	6	29.89	multisynthetase complex p38 auxiliary component
E1BYW9	PSMB3	243	0.14	1.37	3	3	20.00	Proteasome subunit beta type
H9L132	LOC772076	35		1.37	3	3	24.86	Uncharacterized protein
F1NJF0	C1QBP	71	0.25	1.37	3	3	17.55	p32 subunit of splicing factor SF2
P31335	ATIC	212	0.34	1.37	14	14	30.02	Bifunctional purine biosynthesis protein PURH
P43347	TPT1	52	0.61	1.37	4	3	17.44	Translationally-controlled tumor protein homolog
Q5F3J5	PSME3	28	0.24	1.37	7	7	26.38	Proteasome activator complex subunit 3
Q5F3W6	YWHAG	228	0.56	1.37	8	7	21.86	14-3-3 protein gamma
Q5ZKJ2	YWHAH	228	0.56	1.37	8	7	21.05	Uncharacterized protein
Q90593	HSPA5	580	0.49	1.35	16	16	27.15	78 kDa glucose-regulated protein
Q5ZL82	IDH2	156	0.46	1.35	12	12	25.44	Isocitrate dehydrogenase [NADP]
F1P582	UQCRC2	283	0.30	1.34	8	8	20.35	Uncharacterized protein
Q5F491	DDX3X	620	0.70	1.34	14	14	25.96	Putative uncharacterized protein
Q5ZM75	SARS	301	0.46	1.29	13	13	27.63	Putative uncharacterized protein
Q6WNG8	HNRNPH1	345	0.25	1.29	9	9	21.00	Heterogeneous nuclear ribonucleoprotein H1-like protein
Q5ZLI2	PSMA3	93	0.12	1.28	5	5	19.61	Proteasome subunit alpha type
Q90602	HNRNPAB	60	0.22	1.28	6	6	20.20	Single stranded D box binding factor
Q90626	HNRNPAB	46	0.10	1.28	6	6	23.51	Ribonucleoprotein
Q5ZLG0	AACS	113	0.14	1.21	12	12	14.54	Acetoacetyl-CoA synthetase
Q8JG64	PDIA3	196	0.57	1.21	11	11	22.18	Protein disulfide-isomerase A3

Key
emPAI: Exponentially Modified Protein Abundance Index (imported from MASCOT)
emPAI_Calc. Calculated emPAI considering peptides with masses between 700 and 2800 Da.
