# Supplementary material for: The chicken B-cell line DT40 proteome, beadome and interactomes
Source: Data Brief. 2015 Jan 13;3:29–33. doi: 10.1016/j.dib.2014.12.006 (PMC4509924; doi:10.1016/j.dib.2014.12.006)
Supplement: Supplementary file 1 — Supplementary data [file mmc1.zip › Table 3A&B.pdf]

Table 3A. Proteins identified from FLAG pulldowns .

Protein ID	Description	Gene name	Ratios				Log2 ratios				
			DHJLF4	JHDLF4	DHJLF3	JHDLF3	DHJLF4	JHDLF4	DHFLF3	JHDLF3	n
**Q5F356;F1NKL5**	**Phosphatidylinositol-5-phosphate 4-kinase type-2 alpha**	**PIP4K2A**	**0.197**	**7.489**		**1.371**	**-2.340**	**2.905**		0.455	3
**F1NLK4**	**Uncharacterized protein (Fragment)**	**PIP4K2B**	**0.126**	**2.933**		**6.877**	**-2.988**	**1.552**		**2.782**	3
F1NVR9	Uncharacterized protein (Fragment)	KCTD14	0.443	2.913			**-1.175**	**1.543**			2
F1NCR3;Q5ZIZ4	Cytosolic purine 5-nucleotidase	NT5C2	0.300	2.664	0.644	0.173	**-1.736**	**1.413**	-0.635	-2.531	4
F1NHE3;Q5ZKI1	Uncharacterized protein	CLIC2	0.728	2.504			-0.457	**1.324**			2
E1C8Z7	Uncharacterized protein	STK38	0.498	2.161		0.781	**-1.005**	**1.111**		-0.356	3
P11501;F1NVN4;Q5ZKZ3;Q2XQE5;F	Heat shock protein HSP 90-alpha	HSP90AA1	0.664	2.074			-0.591	**1.052**			2
E1C3J7	Uncharacterized protein	BTD	0.615	2.067	0.160	2.023	-0.701	**1.047**	**-2.647**	1.016	4
F1P4H4;Q5ZIM7	Uncharacterized protein	TXNDC5		2.061	0.131	2.084		**1.043**	**-2.929**	1.059	3
B0FLP0;P31335;Q5U784;Q5U785;Q	Aminoimidazole-4-carboxamide ribonucleotide transformylase/IMP	PURH	0.651	1.986			-0.618	**0.990**			2
F1NHI9;Q5ZHU4	6-phosphofructokinase (Fragment)	PFKP	0.534	1.958			**-0.904**	**0.969**			2
F1NGE2;P0CG62;F1NAV4;F1NGE3;C	Ubiquitin (Fragment)	UBB	0.399	1.953			**-1.325**	**0.966**			2
F1NWB7;P08110;Q90WA6;B5AAV6	Endoplasmin	HSP90B1	0.886	1.943			-0.174	**0.958**			2
P00508;F1P180	Aspartate aminotransferase, mitochondrial	GOT2	0.729	1.787	0.452	1.348	-0.455	**0.838**	**-1.145**	0.431	4
Q90593	78 kDa glucose-regulated protein	HSPA5	0.570	1.737	0.667	1.402	**-0.812**	**0.797**	-0.585	0.487	4
F1P5K7;Q5ZMG9	T-complex protein 1 subunit alpha	TCP1	0.517	1.730	1.091		**-0.952**	**0.791**	0.126		3
F1N8F0;Q9YGM2	Delta-9 desaturase	SCD	0.764	1.709	3.001	2.267	-0.388	**0.773**	1.585	1.181	4
E1C3A9;F1NNX1;P81021	Vigilin	HDLBP	0.809	1.647			-0.305	**0.720**			2
F1NW35;E1BTT8;P00340	L-lactate dehydrogenase (Fragment)	LDHA	1.780	1.639	1.095		0.832	**0.713**	0.130		3
F1NT20;E1BV95;Q5ZHQ4	Uncharacterized protein (Fragment)	ACAT2	0.381	1.619			**-1.394**	**0.695**			2
E5DEA6;E5DFI5;E5DFL1;P18944;Q4	Cytochrome c oxidase subunit 2 jabouillei	COX2	0.535	1.596			**-0.902**	**0.674**			2
Q6XD56;F1NNR6	Uncharacterized protein	ILKAP		1.573	2.310			**0.654**	1.208		2
F1P3F9	Glutamate dehydrogenase (Fragment)	GLUD1	0.283	1.553	1.045	0.440	**-1.819**	0.635	0.063	-1.184	4
P15771;F1NU40;F1P336	Nucleolin	NCL	0.559	1.542	1.054	1.691	**-0.840**	0.625	0.075	0.758	4
F1NRW7;F1NWP3;O73885;B3VHV2	Heat shock cognate 71 kDa protein (Fragment)	HSPA8	0.727	1.522			-0.459	0.605			2
Q5F3T2	Uncharacterized protein	SEPT9		1.512	0.832	0.724		0.597	-0.266	-0.466	3
F1NDY9;Q5ZK20	Uncharacterized protein	PDIA4	0.717	1.511	0.478	0.594	-0.481	0.596	**-1.065**	-0.750	4
F1NGA2;F1NI22;Q8UVX3;Q9DDH7;(	ATP synthase subunit alpha (Fragment)	ATP5A1	0.698	1.501			-0.518	0.586			2
P84175	40S ribosomal protein S12	RPS12	1.091	1.491			0.126	0.576			2
Q5ZMU9;F1P4V8;E1BTS8;E1BZ64	Valosin containing protein	vcp	0.688	1.480	1.335	0.155	-0.539	0.565	0.417	-2.690	4
E1C8R1;Q5ZKB9	Probable ATP-dependent RNA helicase DDX6	DDX6	0.537	1.478			**-0.898**	0.564			2
P22451;F2Z4K4	60S ribosomal protein L5	RPL5	0.977	1.460	1.588		-0.034	0.546	0.667		3
F1NZ86;Q5ZM98	Stress-70 protein, mitochondrial	HSPA9	0.781	1.404	0.871	1.209	-0.356	0.489	-0.200	0.274	4
F1NWF6;Q5ZJU3	Asparagine synthetase [glutamine-hydrolyzing]	ASNS	1.085	1.403	0.743	4.324	0.118	0.489	-0.429	**2.112**	4
Q8JFP1;F1NTS2;Q5ZM36;E1BSE5	Eukaryotic initiation factor 4A-II	EIF4A2	0.568	1.390			**-0.815**	0.475			2
F1NY54;F1NEE5;Q5ZMK4	Uncharacterized protein	THRAP3	0.651	1.389	1.937	1.924	-0.619	0.474	0.954	0.944	4
Q5ZLU8;Q45KQ2	Putative uncharacterized protein	RCJMB04_4m1	0.997	1.387			-0.004	0.472			2
Q5ZJJ2	Replication protein A 70 kDa DNA-binding subunit	RPA1	0.784	1.386	0.900	1.541	-0.351	0.471	-0.151	0.624	4

Q5F3L2;F1NYE5;F1NDI3	Putative uncharacterized protein	RCJMB04_14f6	0.631	1.384	0.990	0.841	-0.665	0.469	-0.015	-0.249	4
Q9W7P7;F1NJF0;REV__F1P051	P32 subunit of splicing factor SF2 (Fragment)	SF2	0.809	1.382	0.565		-0.306	0.466	**-0.823**		3
F1NGB1;Q5ZHR1;F1NCP9	Uncharacterized protein	NUCB2	0.979	1.356	1.750	0.297	-0.030	0.439	0.807	-1.751	4
F1NTT0;F1N9T0;P23228	Hydroxymethylglutaryl-CoA synthase, cytoplasmic (Fragment)	HMGCS1	0.834	1.348			-0.261	0.431			2
P00368	Glutamate dehydrogenase 1, mitochondrial	GLUD1	0.619	1.290	0.299	0.510	-0.691	0.368	**-1.740**	-0.972	4
Q90WD0;F1NW48	Actin-related protein 3	ACTR3	0.412	1.290			**-1.278**	0.367			2
P84172	Elongation factor Tu, mitochondrial (Fragment)	TUFM	1.302	1.288	1.480	3.082	0.381	0.366	0.566	**1.624**	4
Q5F491;F1NIX1;F1NIX2	Putative uncharacterized protein	RCJMB04_2a4	0.707	1.286			-0.500	0.363			2
P21868	Casein kinase II subunit alpha	CSNK2A1		1.283	1.175			0.360	0.233		2
F1NXY7;Q98TF8;F1N9J4	60S ribosomal protein L22 (Fragment)	RPL22	0.717	1.276	0.572		-0.480	0.352	**-0.807**		3
F1N914	Uncharacterized protein (Fragment)	PYCR2	1.577	1.272	0.572	1.307	0.657	0.347	**-0.806**	0.386	4
Q5ZL58	Uncharacterized protein	SNRPD3	1.264	1.260	1.231	4.892	0.338	0.333	0.300	**2.290**	4
E1BR89;F1NZ24;Q5ZLZ0	Uncharacterized protein	SLC25A3	0.863	1.248		0.452	-0.212	0.320		-1.145	3
F1NP63	Uncharacterized protein (Fragment)	KPNB1		1.230	2.145	1.130		0.299	1.101	0.177	3
Q5F424	Uncharacterized protein	CCT2	0.870	1.227	1.291	3.498	-0.201	0.295	0.368	**1.807**	4
F2Z4M0;Q9W7I5;Q9I8G9	Histone-binding protein RBBP4	RBBP4	0.661	1.224	1.912	0.504	-0.597	0.291	0.935	-0.987	4
E1BT08;E1C0H5;F1NC26;Q5F3J8	Uncharacterized protein	HSPH1	1.008	1.220			0.012	0.287			2
F1N9X5;Q5ZJC1;F1NPA9;Q6EE58	Uncharacterized protein	RPS3	1.023	1.219	1.069	1.104	0.032	0.285	0.097	0.143	4
Q6EE30	Eukaryotic translation elongation factor 1	eTEF1	0.581	1.211	1.622	0.858	**-0.783**	0.276	0.698	-0.222	4
P18359	Destrin	DSTN	1.138	1.196	0.438	0.569	0.187	0.258	**-1.192**	-0.814	4
Q5ZL34;F1NGU9	Cleavage and polyadenylation specificity factor subunit 6	CPSF6	1.383	1.180	1.468		0.468	0.239	0.554		3
F1NU36;F1NDD6;O57378	Uncharacterized protein (Fragment)	LRPAP1	1.017	1.177		0.344	0.025	0.235		-1.540	3
Q5ZLC5	ATP synthase subunit beta, mitochondrial	ATP5B	0.632	1.176	1.074	0.647	-0.662	0.233	0.103	-0.627	4
O57535;F1N910;F1P3E1;F1NC40	Nucleoside diphosphate kinase	NDK	0.752	1.169			-0.411	0.225			2
F1NKN0;Q5F411	Uncharacterized protein (Fragment)	CCT5	0.642	1.156	1.668	4.373	-0.639	0.209	0.738	**2.129**	4
P00548	Pyruvate kinase muscle isozyme	PKM2	0.640	1.152	1.050	4.959	-0.643	0.204	0.070	**2.310**	4
P08636;F1P597;F1NY38	40S ribosomal protein S17	RPS17	1.000	1.151	0.876		0.000	0.203	-0.191		3
F1NLS0;Q9DEA3	Proliferating cell nuclear antigen (Fragment)	PCNA	0.997	1.151	1.043	0.936	-0.004	0.203	0.061	-0.096	4
Q5ZMD1;Q5ZM05;F1NSY6	14-3-3 protein theta	YWHAQ	0.910	1.147	1.129		-0.135	0.198	0.175		3
F1NHL2	ubiquitin ligase complex	CAND1		1.144	0.472			0.194	**-1.084**		2
F1P010	Uncharacterized protein (Fragment)	ARPC4	0.667	1.141			-0.584	0.190			2
F1NH93	Uncharacterized protein (Fragment)	RPS20	0.907	1.141	0.769	0.509	-0.140	0.190	-0.378	-0.973	4
F1NW43;F1P4U1	Pyruvate kinase (Fragment)	PKM2	0.884	1.140	0.641	3.461	-0.177	0.189	-0.641	**1.791**	4
F1NEV9;P42558	GTP-binding nuclear protein Ran (Fragment)	RAN	1.392	1.136	0.469	0.548	0.477	0.184	**-1.092**	-0.868	4
Q5ZLG7;Q8AYM3;F1NFF6;F1NBW8	Putative uncharacterized protein	RCJMB04_6000	0.943	1.132	0.876		-0.085	0.179	-0.191		3
E1C8Q3	Uncharacterized protein	ALDH18A1		1.131	1.896	3.706		0.178	0.923	**1.890**	3
F1NPG8;Q9I8D6;Q5ZMG3	T-complex protein 1 subunit delta (Fragment)	CCT4	0.674	1.128	1.157	4.729	-0.570	0.173	0.210	**2.242**	4
E1BUZ8	Uncharacterized protein	RPL9	1.101	1.122			0.139	0.167			2
Q5ZJ54;F1NWH9	T-complex protein 1 subunit zeta	CCT6	0.588	1.122		3.707	**-0.765**	0.166		**1.890**	3
F1NBU1	Uncharacterized protein (Fragment)	RPS16	0.887	1.120	1.183	0.948	-0.173	0.163	0.242	-0.077	4

G1K332;F1NCK6;P62801;P70081;Q6	Histone H4 (Fragment)	H4	0.922	1.117	1.447	1.687	-0.117	0.159	0.533	0.755	4
P53488;F1NHN5;F1NRM5	Actin-related protein 2	ACTR2	0.297	1.115	0.404	0.628	**-1.749**	0.157	**-1.309**	-0.671	4
G8JL27;P63247	Guanine nucleotide-binding protein subunit beta-2-like 1 (Fragment	GNB2L1	1.006	1.114	1.184	4.849	0.009	0.155	0.244	**2.278**	4
Q6EE62	Ribosomal protein (Fragment)	RPL10A	0.958	1.101	1.612	1.383	-0.062	0.138	0.689	0.468	4
F1NK96;F1N966;Q5F472	Uncharacterized protein	PDIA6	0.799	1.099	0.521	1.054	-0.323	0.136	**-0.942**	0.076	4
E1C4N0	Uncharacterized protein	RPS10	0.899	1.096	0.449	0.798	-0.154	0.133	**-1.154**	-0.326	4
Q98906	Microtubule-associated protein (Fragment)	MAP4	0.709	1.095			-0.495	0.131			2
Q5ZLX2	Eukaryotic translation initiation factor 2 subunit 1	EIF2S1	1.389	1.093	1.494	3.288	0.474	0.128	0.579	**1.717**	4
E1BZG4	Uncharacterized protein	EEF1D	1.165	1.089	1.035		0.220	0.122	0.049		3
F1NU17;P51903	Phosphoglycerate kinase	PGK1	0.920	1.078	0.368	0.827	-0.120	0.108	**-1.441**	-0.275	4
P47826;F1NB66	60S acidic ribosomal protein P0	RPLP0	0.972	1.075	1.128	2.443	-0.041	0.105	0.174	1.289	4
Q5ZL82	Uncharacterized protein	HSDL2		1.072	0.721			0.100	-0.472		2
F1NVF0;Q5ZL51	Uncharacterized protein	VPS35	0.874	1.071	1.538	0.916	-0.195	0.098	0.621	-0.127	4
Q9IAY5	Protein syndesmos	SDOS	0.840	1.070	0.983	0.595	-0.251	0.097	-0.025	-0.750	4
Q5ZL72	60 kDa heat shock protein, mitochondrial	HSPD1	0.544	1.068		1.562	**-0.878**	0.095		0.643	3
F1NC02	Proteasome subunit alpha type (Fragment)	PSMA4	1.809	1.064		2.831	0.855	0.090		**1.501**	3
Q5ZLR5	Cytochrome b-c1 complex subunit Rieske, mitochondrial	UQCRFS1	0.978	1.064	2.269	2.915	-0.032	0.090	1.182	**1.544**	4
Q5ZKM2;Q90835;F1N9H4;Q6PTX1	Elongation factor 1-alpha	EEFIA1	0.749	1.057	0.504	1.074	-0.417	0.080	**-0.989**	0.102	4
F1NK29;Q5ZM14	Na(+)/H(+) exchange regulatory cofactor NHE-RF1	SLC9A3R1	0.611	1.057			-0.710	0.079			2
E1BXS5	Uncharacterized protein	DECR1	1.662	1.055	0.971	1.770	0.733	0.077	-0.043	0.824	4
E1C4M0	Uncharacterized protein	RPS2	0.969	1.053	1.254	2.095	-0.046	0.074	0.326	1.067	4
Q90679	Thymocyte nuclear protein 1	THYN1	0.779	1.053	0.688	0.736	-0.361	0.074	-0.539	-0.442	4
E1BV44	Uncharacterized protein (exportin)	CSE1L	0.571	1.050	0.721	0.760	**-0.807**	0.070	-0.471	-0.396	4
F1NCI4;Q5ZJ56;F1NCI6	60S ribosomal protein L7 (Fragment)	RPL7	0.846	1.046	1.345		-0.242	0.064	0.427		3
F1NU56	Uncharacterized protein (Fragment)	RPS25	1.019	1.041	0.777		0.027	0.058	-0.364		3
F1P1A8	Uncharacterized protein (Fragment)	SMARCC1		1.041	1.207	0.810		0.058	0.271	-0.304	3
Q5ZKC9;F1NPX9;Q9PS14	14-3-3 protein zeta	YWHAZ	0.808	1.034	1.263		-0.307	0.048	0.337		3
F1NV05;F1NIJ6;Q5ZMU3;CON__Q3	Glucose-6-phosphate isomerase (Fragment)	GPI	1.059	1.028		1.077	0.083	0.039		0.107	3
Q5ZLH1	Uncharacterized protein	RPA2	1.168	1.026	0.426	1.449	0.224	0.037	**-1.231**	0.535	4
E1C8W4	Ubiquitin carboxyl-terminal hydrolase	USP5		1.013	0.888	0.503		0.019	-0.171	-0.990	3
E1BSD8	Uncharacterized protein	POLR2H	0.639	1.008	1.530		-0.647	0.011	0.614		3
F1NLM5	Uncharacterized protein (Fragment)	SNRPD1	0.768	1.005	0.965		-0.382	0.007	-0.052		3
Q5ZIT7;F1P3L9;F1NDQ1	Putative uncharacterized protein	RCJMB04_23k2/PSMC3		1.002	1.376	1.035		0.003	0.461	0.050	3
E1BTG1	Uncharacterized protein	RPL12	1.333	0.993	0.786	1.986	0.414	-0.011	-0.348	0.990	4
F1NW84;F1NVA4;P16039;Q6LEK3;F	Nucleophosmin (Fragment)	NPM1	0.916	0.991			-0.126	-0.013			2
B0LVF9;B0LVG0;F1NUN1	Microtubule-associated protein	MAPT	0.925	0.989			-0.113	-0.016			2
F1P304	Uncharacterized protein (Fragment)	ATP5O	0.799	0.989	1.409		-0.323	-0.016	0.495		3
F1P1A5	Uncharacterized protein (Fragment)	TKT	1.057	0.987	0.758	4.713	0.080	-0.019	-0.399	**2.237**	4
F1NBD7;P13863;F1NA68;A2IAR9;B6	Uncharacterized protein	CDC2	1.039	0.986	1.038	1.768	0.055	-0.020	0.054	0.822	4
P00356;F1NH87	Glyceraldehyde-3-phosphate dehydrogenase (Fragment)	GAPDH		0.978	0.322	1.451		-0.032	**-1.637**	0.537	3

D0EKR3	Peptidyl-prolyl cis-trans isomerase	PPCTI	0.740	0.976	0.182	2.071	-0.435	-0.035	**-2.458**	1.050	4
Q5ZIZ5	Uncharacterized protein	NONO	1.144	0.966	2.834	1.516	0.194	-0.050	1.503	0.600	4
F1NN16	Uncharacterized protein (Fragment)	RPS7	0.943	0.966	0.663	0.362	-0.085	-0.050	-0.592	-1.467	4
Q5ZJK8;F1NK38;F1NZN1	T-complex protein 1 subunit eta	CCT7	0.537	0.957		3.234	**-0.898**	-0.063		**1.693**	3
Q98TH5;F1N9W9;F1P553	Ribosomal protein S11	cRPS11	0.928	0.956	1.588		-0.107	-0.065	0.667		3
F1NB02;Q6EE57	40S ribosomal protein S8 (Fragment)	RPS8	1.012	0.955	1.665	2.717	0.017	-0.066	0.736	1.442	4
F1N8Z4;Q5ZIC4	Uncharacterized protein	RUVBL1		0.949	1.283	3.515		-0.076	0.360	**1.814**	3
F1NRB9;P09645;F1NUP6	Uncharacterized protein (Fragment)	LONP1		0.944	1.525			-0.083	0.609		2
P47836;F1NFC6;F1P0F2;REV__F1NN	40S ribosomal protein S4	RPS4	0.945	0.944	0.949	0.554	-0.082	-0.083	-0.075	-0.851	4
Q5ZMQ2;Q5ZLX9	Actin, cytoplasmic 2	ACTG1	0.462	0.943			**-1.115**	-0.084			2
E1BYM1	mRNA cap guanine-N7 methyltransferase	RNMT	1.035	0.940	1.303	0.525	0.049	-0.089	0.382	-0.930	4
Q5ZHW8	Uncharacterized protein	RPS14	0.776	0.935	1.303	0.473	-0.365	-0.096	0.382	-1.081	4
F1P1K3	Uncharacterized protein (Fragment)	ARPC2	0.674	0.935		0.730	-0.570	-0.097		-0.455	3
E1BXA2;P13127;Q5ZLP3	F-actin-capping protein subunit alpha-1	CAPZA1	0.473	0.932			**-1.080**	-0.101			2
F1NTC7;Q98TF9	Uncharacterized protein (Fragment)	RPL14	1.028	0.930	1.580		0.040	-0.105	0.660		3
F1NDC2	Uncharacterized protein (Fragment)	RPS23	0.875	0.928	2.276		-0.192	-0.108	1.186		3
Q6ITC7;F1NGB4;REV__E1C1R6	40S ribosomal protein S13	RPS13	0.895	0.926	1.358		-0.159	-0.111	0.441		3
Q5ZKA5	Bifunctional methylenetetrahydrofolate dehydrogenase/cyclohydro	MTHFD2		0.918	0.658	1.087		-0.124	-0.603	0.120	3
E1C8F7	Uncharacterized protein	RPL24	0.818	0.907	0.746		-0.289	-0.142	-0.423		3
E1C658	Uncharacterized protein	ATP5H	0.501	0.900	1.528		**-0.996**	-0.152	0.612		3
F1NPD3	Uncharacterized protein (Fragment)	RPL18A	1.000	0.898	2.660		0.000	-0.155	1.411		3
F1NIX0	Uncharacterized protein (Fragment)	RPL8	1.102	0.891	0.968		0.140	-0.166	-0.047		3
Q5ZLN1;F1NHM9;F1NQ41	Phosphoglycerate mutase 1	PGAM1	0.529	0.888	0.300	3.337	**-0.920**	-0.171	**-1.736**	**1.739**	4
D0VX26	Mitochondrial cytochrome c1, heme protein	MCC1	0.929	0.888	1.718	0.999	-0.106	-0.172	0.781	-0.001	4
Q90602;F1NIB2;F1NIB4;Q90626	Single stranded D box binding factor	HNRNPAB	0.697	0.884	0.953	1.691	-0.520	-0.178	-0.070	0.758	4
G1K326;P0CB50	Peroxiredoxin-1 (Fragment)	PRDX1	0.944	0.879	2.206	1.941	-0.083	-0.186	1.141	0.957	4
F1P4V6	Uncharacterized protein (Fragment)	RPL31	1.003	0.872	1.014		0.004	-0.198	0.020		3
F1N941;E1C0S2;Q5ZK88	Paraspeckle component 1	PSPC1		0.870	2.130	2.372		-0.201	1.091	1.246	3
Q5F4A4;E1C707;Q5ZLI1;F1N808	Inosine-5-monophosphate dehydrogenase	IMPDH2	1.128	0.869	0.647	0.925	0.173	-0.202	-0.629	-0.113	4
E1C1X6	Uncharacterized protein	MOB2	0.886	0.859			-0.175	-0.220			2
E1C2C3	Uncharacterized protein	SF3B1		0.859	1.505	5.177		-0.220	0.589	**2.372**	3
Q5F426	Uncharacterized protein	PDHA1		0.856	1.199	0.878		-0.225	0.262	-0.188	3
Q5ZK62;F1NG96;F1NGZ5	Arf-GAP with coiled-coil, ANK repeat and PH domain-containing prot	ACAP2	0.658	0.852		0.155	-0.604	-0.231		-2.691	3
P00940	Triosephosphate isomerase	TPI1	1.022	0.851	0.339		0.031	-0.233	**-1.561**		3
F1NXW3	Uncharacterized protein (Fragment)	RPS15A	1.059	0.848	0.724		0.083	-0.237	-0.465		3
F1P555;Q91017	Uncharacterized protein	SFPQ	0.921	0.848	1.959	1.813	-0.118	-0.238	0.970	0.858	4
Q90WG1;Q90WG2;F1NMV1;F1NCT	Spindlin-Z	SPINZ	1.524	0.843		0.666	0.608	-0.246		-0.587	3
P00337	L-lactate dehydrogenase B chain	LDHB	0.583	0.836	0.220	4.532	**-0.779**	-0.259	**-2.185**	**2.180**	4
F1NEG6;F1NJJ5;Q6WNG8;F1NJJ4;Q	Uncharacterized protein	HNRNPH1	0.919	0.828		4.256	-0.122	-0.272		**2.090**	3
P67883;G1K316	60S ribosomal protein L30	RPL30	0.844	0.822	0.548		-0.245	-0.283	**-0.869**		3

E1BSL7	Uncharacterized protein	ERP44		0.818	1.206	0.794		-0.289	0.270	-0.332	3
E1BSS3	Uncharacterized protein	EIF3A	1.321	0.814		0.279	0.402	-0.298		-1.841	3
F1NUY0;F1NUX9	Uncharacterized protein (Fragment)	C2orf56	0.472	0.804			**-1.082**	-0.315			2
F1NHA8;P32429;F2Z4L5	60S ribosomal protein L7a (Fragment)	RPL7A	0.970	0.799			-0.043	-0.324			2
P09244	Tubulin beta-7 chain	TUBB7	1.315	0.798	1.348		0.395	-0.325	0.430		3
Q5F3D2	Uncharacterized protein	HNRNPH3	1.016	0.789	1.102	2.367	0.023	-0.341	0.139	1.243	4
F1NQG5;F1P118;P51417	Ribosomal protein L15 (Fragment)	RPL15	0.950	0.784	1.474		-0.074	-0.350	0.560		3
Q5ZHZ0	Spliceosome RNA helicase DDX39B	DDX39B		0.771	0.982	0.606		-0.376	-0.026	-0.722	3
P50890;Q6SVA6;F1NKH1	40S ribosomal protein SA	RPSA	1.137	0.759	0.631	0.888	0.185	-0.398	-0.664	-0.172	4
E1BXK5;Q5F3M2	Uncharacterized protein	RBM5	1.348	0.756		0.524	0.431	-0.404		-0.933	3
F1NX13;Q5H7M6	Uncharacterized protein	DRG1		0.724	0.869	1.576		-0.466	-0.203	0.656	3
Q5ZLE6	Eukaryotic translation initiation factor 3 subunit H	EIF3H	1.100	0.706	0.927		0.137	-0.501	-0.110		3
F1ND80;Q5F428	Uncharacterized protein (Fragment)	EIF3EIP	0.844	0.701	3.119		-0.245	-0.513	1.641		3
F1NL05;P26446;F1P5K6;F1NL24;Q9	Poly [ADP-ribose] polymerase 1	PARP1	2.145	0.685	2.026		1.101	-0.546	1.019		3
O92956;A2SXN8;B1Q2X9;E2G6M3;I	Avian RSVSB Gag-Pro-Pol polyprotein	RAV-0;SE21Q1b;	1.149	0.608			0.200	-0.718			2
F1NZE2;Q5ZJ64	Eukaryotic translation initiation factor 3 subunit M (Fragment)	EIF3M	1.190	0.603	1.673	0.812	0.251	-0.731	0.742	-0.301	4
E1BZE6;F1NBB6;F1P5I0	Uncharacterized protein	HNRNPA3		0.603	3.051	3.155		-0.731	1.609	**1.658**	3
Q5F3C0;E1BVJ5	Putative uncharacterized protein	RCJMB04_22h1		0.540	2.926	1.889		-0.888	1.549	0.918	3
E1C2E1;E1C2E2;F1P1V1;Q5ZLR3	Uncharacterized protein	STK38L	1.494	0.485			0.579	-1.045			2
E1BYN9	Uncharacterized protein	PRPSAP1	4.118	0.447			2.042	-1.162			2
F1NCM5;Q5ZII4	Uncharacterized protein	LTB4DH	2.216	0.443		0.845	1.148	-1.174		-0.242	3
E1C6D1;E1BXF5	Microtubule-associated protein	MAP2	2.079	0.413	1.784		1.056	-1.276	0.835		3
F1NZC6;Q5ZL26	Phosphoribosyl pyrophosphate synthase-associated protein 2	PRPSAP2	3.411	0.184			1.770	-2.441			2
P14105;F1NCD4;F1NXC9;Q7LZ83	Myosin-9	MYH9	0.517		0.957		**-0.953**		-0.063		2
F1NIN4;P24032;P02612;E1C6R9	Uncharacterized protein (Fragment)	MYL12A	0.561		1.401		**-0.834**		0.487		2
Q5ZL53;F1NH81;F1NH82;E1BWJ7;E	Putative uncharacterized protein	RCJMB04_7l20	0.615			0.670	-0.701			-0.577	2
P12276-2;E1BW07;F1N8A8;P12276	Isoform 1 of Fatty acid synthase	FASN	0.667		2.124		-0.585		1.086		2
Q5ZMN1;E1C769	Uncharacterized protein	G3BP1	0.710		2.067		-0.494		1.047		2
F1NXA5;Q5ZI60;Q5ZM02;Q5ZLS1;F1	Uncharacterized protein (Fragment)	CORO1C	0.749		0.944		-0.416		-0.083		2
F1NDI7;F1NC33;Q04619	Heat shock cognate protein HSP 90-beta (Fragment)	HSP90AB1	0.808		0.704	0.573	-0.307		-0.507	-0.803	3
F1NHF1	Uncharacterized protein (Fragment)	ATP5J2	0.819		0.787		-0.288		-0.345		2
Q5ZMN3;F1N833;F1NPZ8	Prohibitin-2	PHB2	0.859		1.429	1.116	-0.219		0.515	0.159	3
G1K331;G1K329;P84229;C6ZL36;Q0	Histone H3 (Fragment)	LOC417953	0.870			1.599	-0.202			0.677	2
Q5ZHY1;F1NYA9;Q9YGQ1	Putative uncharacterized protein	RCJMB04_32c11	0.905		0.721		-0.144		-0.472		2
F1NLT9;F1NCL1;F1NDS3;P02263;Q9	Histone H2A (Fragment)	HIST2H2AC	0.908			3.209	-0.139			**1.682**	2
Q09121;Q07460;G1K322	Eukaryotic translation initiation factor 5A-1	EIF5A1	0.921		0.548		-0.119		**-0.868**		2
F1NSP8;F1NM96;Q2MHD2	Uncharacterized protein (Fragment)	HNRNPU	0.959		2.498	1.216	-0.061		1.321	0.282	3
P61355	60S ribosomal protein L27	RPL27	0.975		1.637		-0.036		0.711		2
P02272;Q5ZMD6;F2Z4M5	Histone H2A.V	H2AFV	0.976		0.785	1.103	-0.035		-0.350	0.141	3
E1BW78	Uncharacterized protein	COX6C	0.982		1.905		-0.026		0.930		2

E1BS06	Uncharacterized protein	RPL23A	0.987		0.611		-0.019		-0.711		2
F2Z4K7;Q5ZK27	Uncharacterized protein	RPS3A	1.000		1.269		-0.001		0.344		2
A3RL83;Q5ZMN0	Lectin-associated matrix protein (Fragment)	hLAMP-1	1.034			1.509	0.048			0.594	2
F1NNC3;P62846;F2Z4M3	40S ribosomal protein S15 (Fragment)	RPS15	1.068		3.046		0.095		1.607		2
P62207;Q5ZL39;Q9PSQ6	Serine/threonine-protein phosphatase PP1-beta catalytic subunit	PPP1CB	1.069			1.854	0.097			0.891	2
F1NG53;Q6EE61	Uncharacterized protein (Fragment)	RPL17L	1.073		1.206		0.102		0.271		2
F1NP85	Uncharacterized protein (Fragment)	RPL21	1.080		1.046		0.111		0.065		2
P0C1H5;F1NF30	Histone H2B 7	H2B-VII	1.118		1.692		0.161		0.759		2
F1NE72;Q5ZKP8;E1C3E0;Q8JGU2;RI	Lysyl-tRNA synthetase	KARS	1.164		2.303		0.219		1.203		2
F1P179;Q5ZJ86	Uncharacterized protein	EPRS	1.172		1.116		0.229		0.159		2
E1BW50;Q5ZHU8	Uncharacterized protein	CTNNBL1	1.175		1.839		0.232		0.879		2
Q9PV94	Small nuclear ribonucleoprotein-associated protein B	SNRPB	1.181		1.046	1.339	0.241		0.065	0.421	3
F1P0E4;Q9I9D1;E1BYN7	Uncharacterized protein (Fragment)	VDAC2	1.186		0.973	2.975	0.246		-0.040	**1.573**	3
Q5ZME1	Uncharacterized protein	HNRNPA2B1	1.186		2.267	3.067	0.246		1.181	**1.617**	3
E1C2L5	Uncharacterized protein	THOC4	1.193		0.736	0.358	0.254		-0.443	-1.484	3
F1N9U8;Q5ZIX2;E1BZW5	Uncharacterized protein	ETFA	1.197		0.620	0.769	0.259		-0.689	-0.380	3
Q5ZM66	Putative uncharacterized protein	RCJMB04_2p17	1.225		0.579		0.292		**-0.788**		2
F1NG68	Uncharacterized protein (Fragment)	DECR2	1.274			0.287	0.349			-1.799	2
Q5ZMT0;F1NGW3;F1P2P9;F1P2Q0	14-3-3 protein epsilon	YWHAE	1.295			0.906	0.373			-0.143	2
E1BV75	Uncharacterized protein	DHRS7	2.512		2.667	4.595	1.329		1.415	**2.200**	3
						median	-0.138	0.192	-0.025	0.107	
						1sd	0.6059	0.5403	0.8619	1.1866	
						med+/-	-0.701	2.090	0.282	2.076	

Key: **Bold** indicates ratio +/- 1sd of median. Heatmap annotation identifies enriched high confidence ratios (green) decreasing to low confidence or contradictory ratios (red). Green proteins are those also found in Talon pulldowns

Table 3B. Proteins identified from TALON pulldowns .

			Ratios				Log2 ratios				
Protein IDs	Description	Gene	DHJLT4	JHDLT4	DHJLT3	JHDLT3	DHJLT4	JHDLT4	DHJLT3	JHDLT3	n
Q98TX7;Q98TX6	Interferon regulatory factor 4	IRF-4	0.172	3.794	0.158	14.800	**-2.536**	**1.924**	**-2.660**	**3.888**	4
D0EL81	Glutathione reductase (Fragment)	GR	0.260		0.154	8.186	**-1.943**		**-2.700**	**3.033**	3
F1NQF4;E0A2T5;F1NC96;P14791;Q(	Heme oxygenase 1 (Fragment)	HMOX1;hmox1	0.342	3.752	0.139	4.578	**-1.546**	**1.908**	**-2.843**	**2.195**	4
E1BYG9;E1BYG7;Q5ZM86	Coiled-coil domain-containing protein 50	CCDC50	0.370	1.500			**-1.434**	**0.585**			2
P00337	L-lactate dehydrogenase B chain	LDHB	0.435	1.171	3.339	0.161	**-1.202**	0.228	1.739	-2.638	4
F1NFD7;Q9YGC1;Q9YGC1-2	B-cell linker protein (Fragment)	BLNK	0.463	1.910	0.303	5.029	**-1.111**	**0.933**	**-1.723**	**2.330**	4
**Q5F356;F1NKL5;F1NLK4**	**Phosphatidylinositol 5-phosphate 4-kinase type-2 alpha**	**PIP4K2A;PIP4K2**	**0.469**		**0.606**	**1.516**	**-1.091**		**-0.722**	**0.601**	3
E1C540	Uncharacterized protein	NPM3	0.496	1.945			**-1.011**	**0.960**			2
E1C0Q5	Uncharacterized protein	ACAT1	0.502		0.731		**-0.993**		-0.451		2
F1P3F9	Glutamate dehydrogenase (Fragment)	GLUD1	0.530	1.921	0.174	3.350	**-0.917**	**0.942**	**-2.521**	**1.744**	4
F1P3B8;Q5F3U9;E1BT16	Sister chromatid cohesion protein PDS5 homolog B	PDS5B	0.532	1.095	4.137	0.658	**-0.910**	0.130	2.049	-0.604	4
F1NCW5;F1P3D9;Q5ZIF4;F1NWI3;F	Uncharacterized protein (Fragment)	SLC25A13;RCJMI	0.554			0.827	**-0.851**			-0.274	2
E1C3D2;E1BX15;Q5ZMH1	Uncharacterized protein	Sep-02;SEPT2	0.555	1.520			**-0.848**	**0.604**			2

F1NTT0;F1N9T0;P23228	Hydroxymethylglutaryl-CoA synthase, cytoplasmic (Fragment)	HMGCS1	0.556	2.195		1.748	**-0.847**	**1.134**		0.806	3
F1NDY9;Q5ZK20	Uncharacterized protein	PDIA4;RCJMB04_	0.559	1.543	0.241	2.249	**-0.839**	**0.626**	**-2.055**	**1.169**	4
Q98906	Microtubule-associated protein (Fragment)	MAP4	0.564	1.212	0.578	3.822	**-0.825**	0.277	**-0.791**	**1.934**	4
P00368	Glutamate dehydrogenase 1, mitochondrial	GLUD1	0.565	1.936	0.211	3.286	**-0.824**	**0.953**	**-2.242**	**1.716**	4
F1NCS6	Uncharacterized protein (Fragment)	MTHFD1L	0.565	2.240	2.042	1.285	**-0.822**	**1.164**	1.030	0.362	4
Q5ZM14;F1NK29	Na(+)/H(+) exchange regulatory cofactor NHE-RF1	SLC9A3R1	0.570	1.301	0.193	3.261	**-0.810**	0.379	**-2.371**	**1.705**	4
Q5F3D7	U3 small nucleolar RNA-associated protein 15 homolog	UTP15	0.573		2.793		**-0.803**		1.482		2
F1NCR3;Q5ZIZ4	Cytolic purine 5-nucleotidase	NT5C2	0.585	1.881			**-0.774**	**0.911**			2
A7UEA8;F1P3I8;P28173	Amidophphoribyltransferase	GPAT;PPAT	0.588	1.323			**-0.766**	0.404			2
P21868	Casein kinase II subunit alpha	CSNK2A1	0.592	1.298			**-0.756**	0.376			2
Q5F3T3;F1NIP9;E1BVH4;F1NIL0;F1N	Putative uncharacterized protein	RCJMB04_7k16;S	0.595	1.417	1.456		**-0.750**	0.503	0.542		3
E1BXT3;F1NUM3;Q5ZKZ1	Uncharacterized protein	GOSR2;RCJMB04	0.621	1.209			**-0.687**	0.273			2
F1NNS8	Uncharacterized protein (Fragment)	PRDX4	0.624	1.520	0.461	3.702	**-0.679**	**0.604**	**-1.116**	**1.888**	4
F1P3F1	Adenylhomocysteinase (Fragment)	AHCY	0.627	1.631	3.903	0.504	**-0.674**	**0.706**	1.965	-0.988	4
Q07460;G1K322	Eukaryotic translation initiation factor 5A-2	EIF5A2	0.636	1.372	0.574	2.292	**-0.652**	0.456	**-0.801**	**1.197**	4
Q5ZMH2;F1NFE2;F1NID2;Q5ZHK0;F	Putative uncharacterized protein	RCJMB04_2a19	0.638	1.482	4.404	0.469	**-0.648**	**0.568**	2.139	-1.092	4
F1NPF3	Uncharacterized protein (Fragment)	NDUFB9	0.655	1.475			**-0.611**	**0.561**			2
Q5ZMG9;F1P5K7	T-complex protein 1 subunit alpha	RCJMB04_2b5;T	0.664		3.446		**-0.591**		1.785		2
G1K330;P70082;F1NDT5;Q92064;F1	Histone H2A (Fragment)	H2A-IX	0.672	1.066	3.649	0.387	**-0.572**	0.092	1.867	-1.368	4
Q5ZJ47;F1NWA8	Putative uncharacterized protein	RCJMB04_20m7	0.678	0.942	3.890		**-0.561**	-0.087	1.960		3
F1NMC3;Q5ZI76	Uncharacterized protein	MTHFD1;RCJMB	0.681	1.436	2.576	0.826	-0.554	0.522	1.365	-0.276	4
Q5F4B2;F1N8E4	Switch-associated protein 70	SWAP70	0.695	1.395		0.673	-0.526	0.480		-0.572	3
E1BSI5	Uncharacterized protein	THOC2	0.698	0.790	3.177	0.502	-0.519	-0.340	1.668	-0.994	4
F1NBY8;Q5ZLG0;F1NFH5;Q5ZHT4;Q	Acetoacetyl-CoA synthetase	AACS	0.701	0.935	0.491	1.202	-0.513	-0.096	**-1.027**	0.265	4
F1NPV3;Q9W719;F1NBZ6	Hypoxanthine-guanine phphoribyltransferase (Fragment)	HPRT1	0.703	1.848			-0.508	**0.886**			2
Q5ZJ54;F1NWH9	T-complex protein 1 subunit zeta	CCT6	0.710	1.127		0.705	-0.494	0.172		-0.504	3
Q5ZIZ0	6-phphogluconate dehydrogenase, decarboxylating	PGD	0.715	1.400	0.234	0.891	-0.484	0.485	**-2.096**	-0.166	4
Q9W7P7;F1NJF0	P32 subunit of splicing factor SF2	SF2;C1QBP	0.716	1.365	0.785	1.400	-0.482	0.449	-0.349	0.485	4
F1NLT9;F1NCL1;F1NDS3;P02263;Q9	Histone H2A (Fragment)	HIST2H2AC;H2A-	0.721	1.092		0.465	-0.471	0.128		-1.106	3
F1P304	Uncharacterized protein (Fragment)	ATP5O	0.729	1.030			-0.457	0.043			2
E1C6U8;F1NXR8;Q5ZIU4	Uncharacterized protein	LUC7L3;RCJMB0	0.734		3.297	0.403	-0.447		1.721	-1.311	3
Q90997;F1NWR2;F1NTM6	Transferrin receptor protein 1	TFRC	0.735	2.052			-0.445	**1.037**			2
F1P0L8;Q0KKP4;Q0KKP5	Uncharacterized protein (Fragment)	CYP51A1;CYP51	0.741	2.133	1.697	0.695	-0.432	**1.093**	0.763	-0.525	4
Q90593	78 kDa gluce-regulated protein	HSPA5	0.743	1.256	0.672	2.723	-0.429	0.329	**-0.573**	**1.445**	4
F1NY37;Q5ZM10	Acyl-coenzyme A oxidase	ACOX1;RCJMB04	0.746	1.431	0.407	1.462	-0.422	**0.517**	**-1.298**	0.548	4
Q5ZJB8;F1NTL1	Putative uncharacterized protein	RCJMB04_19g20	0.747	1.149	2.838		-0.420	0.200	1.505		3
F1P529;F1NZF7;Q5ZI54	Uncharacterized protein	SF3B3	0.748	0.891			-0.419	-0.167			2
Q5F3T2	Uncharacterized protein	SEPT9	0.752	1.112		0.323	-0.411	0.153		-1.628	3
P31335;Q5U784;Q5U785;Q5XKY5;B	Bifunctional purine biynthesis protein PURH	ATIC;PURH	0.755	2.932	0.276		-0.405	**1.552**	**-1.860**		3
F1NWB7;P08110;B5AAV6;Q90WA6	Endoplasmin	HSP90B1	0.756	1.129	0.246	1.204	-0.404	0.175	**-2.023**	0.267	4

E1C4N0	Uncharacterized protein	RPS10	0.759	1.130	1.595	0.548	-0.399	0.177	0.674	-0.869	4
F1NUN1	Microtubule-associated protein (Fragment)	MAP	0.765	0.924			-0.387	-0.115			2
D2Z1L9;F1NJF3	LIM and SH3 protein 1	LASP1	0.775	0.977			-0.368	-0.034			2
E1C8W4	Ubiquitin carboxyl-terminal hydrolase	USP5	0.776	0.746	0.364	0.603	-0.366	-0.422	**-1.460**	-0.730	4
F1NBW0	Uncharacterized protein (Fragment)	MTPAP	0.778	1.162	4.106		-0.363	0.216	2.038		3
F1NB38	Ethylmalonyl-CoA decarboxylase	ECHDC1	0.779	1.433		1.406	-0.360	0.519		0.492	3
E1BUW6	Microtubule-associated protein	MAP4	0.791	1.226	0.329	2.999	-0.338	0.294	**-1.604**	**1.584**	4
Q5ZKA5	Bifunctional methylenetetrahydrofolate dehydrogenase/cyclohydro	MTHFD2	0.795	1.052	0.741	1.262	-0.330	0.073	-0.433	0.336	4
E1BUP6	Uncharacterized protein	PDIA5	0.797	1.039	0.389	1.352	-0.327	0.055	**-1.363**	0.435	4
F1NHI9;Q5ZHU4;Q90YA3	6-phphofructokinase (Fragment)	PFKP	0.801	1.183	2.315		-0.319	0.242	1.211		3
E1BT94	Uncharacterized protein	NDUFB6	0.803	1.202			-0.316	0.265			2
F1NPG8;Q9I8D6;Q5ZMG3	T-complex protein 1 subunit delta (Fragment)	CCT4;tcp-1 delta	0.804	0.780	3.221	0.878	-0.316	-0.359	1.687	-0.187	4
Q5ZKB9;E1C8R1	Probable ATP-dependent RNA helicase DDX6	DDX6	0.806	1.008	3.016	0.228	-0.311	0.011	1.593	-2.136	4
F1NSC1	Uncharacterized protein (Fragment)	ATP5F1	0.811	0.926			-0.303	-0.111			2
D0VX26	Mitochondrial cytochrome c1, heme protein	MCC1	0.812	0.804	0.569	1.274	-0.300	-0.315	**-0.813**	0.349	4
F1NPF0;Q5ZMS3	Eukaryotic translation initiation factor 2 subunit 3	EIF2S3	0.815	1.206	3.371	0.192	-0.295	0.270	1.753	-2.378	4
F1NY29;Q5F3D8	Uncharacterized protein	ETFDH;RCJMB04	0.817		2.168		-0.292		1.116		2
F1NGU3;Q5ZIN9;F1NX33	Uncharacterized protein (Fragment)	LRPPRC	0.821	1.167	1.227	0.969	-0.284	0.223	0.295	-0.046	4
E1BS67	Serine hydroxymethyltransferase	SHMT1	0.825	0.980	1.955	1.552	-0.277	-0.029	0.967	0.634	4
F1NGP4	Uncharacterized protein (Fragment)	DUS3L	0.831	1.037		0.274	-0.267	0.053		-1.869	3
F1NY54;F1NEE5;Q5ZMK4	Uncharacterized protein	THRAP3;RCJMB0	0.832	0.986	3.471	1.898	-0.265	-0.021	1.795	**0.924**	4
P47836;F1NFC6;F1P0F2	40S ribomal protein S4	RPS4	0.836	1.034	1.609	0.362	-0.258	0.048	0.686	-1.468	4
P00508;F1P180	Aspartate aminotransferase, mitochondrial	GOT2	0.845	1.731	0.836	1.190	-0.244	**0.791**	-0.258	0.251	4
F1NH93	Uncharacterized protein (Fragment)	RPS20	0.849	1.328			-0.236	0.409			2
E1BZ79	Uncharacterized protein	MVK	0.849	1.017			-0.236	0.024			2
F1NEI4;F1NA45;Q90706	Uncharacterized protein	C5H14orf166;CLI	0.851		3.107		-0.233		1.636		2
F1NBI2	Uroporphyrinogen decarboxylase (Fragment)	UROD	0.851	1.197	1.914	1.181	-0.232	0.260	0.937	0.240	4
E1C3T6	Uncharacterized protein	ECI1	0.852	1.115	0.404	2.313	-0.231	0.157	**-1.308**	**1.210**	4
F1NKN0;Q5F411	Uncharacterized protein (Fragment)	CCT5	0.853	0.687	4.634	0.587	-0.229	-0.541	2.212	-0.769	4
P02272;Q5ZMD6;F2Z4M5;E1C4W6;	Histone H2A.V	H2AFV;H2AFZ	0.855	0.688		0.482	-0.226	-0.540		-1.054	3
Q90705	Elongation factor 2	EEF2	0.856	1.094	0.152	2.083	-0.225	0.130	**-2.715**	**1.059**	4
E1BU15;F1NRN7;Q5F4C4	Leucine-rich repeat protein SHOC-2	SHOC2	0.857	0.630			-0.223	-0.667			2
Q09121	Eukaryotic translation initiation factor 5A-1	EIF5A1	0.858	1.158	0.577	2.031	-0.221	0.212	**-0.793**	**1.022**	4
F1P4F4;Q5ZM63	Uncharacterized protein	SSR1;RCJMB04_	0.859		2.610	1.081	-0.220		1.384	0.112	3
F1NS60	Uncharacterized protein (Fragment)	MMS19	0.862	1.044	2.275	0.730	-0.215	0.063	1.186	-0.454	4
F1NGB1;Q5ZHR1;F1NCP9	Uncharacterized protein	NUCB2;RCJMB04	0.862	1.325	0.308	3.064	-0.215	0.405	**-1.698**	**1.615**	4
F1NC26;Q5F3J8	Uncharacterized protein	HSPA4L;RCJMB0	0.865	1.010	0.517	0.633	-0.209	0.014	**-0.952**	-0.661	4
F1NW84;F1NVA4;P16039;Q6LEK3;F	Nucleophmin (Fragment)	NPM1	0.868	1.001	0.810		-0.204	0.001	-0.305		3
E1BX03;F1NGC0;Q5ZM76;F1NYJ3	Uncharacterized protein	FUBP1	0.868	1.041		3.135	-0.204	0.058		**1.649**	3
F1NCC9;Q5ZLP0	Uncharacterized protein	SMARCE1;RCJMI	0.872	1.087	0.873	0.718	-0.198	0.121	-0.195	-0.479	4

E1BV85	Uncharacterized protein	LRRC20	0.873	0.704			-0.196	-0.507			2
F1NBU1	Uncharacterized protein (Fragment)	RPS16	0.874	1.278			-0.195	0.354			2
E1BX21	Uncharacterized protein	SIN3A	0.880	1.197	4.561		-0.184	0.260	2.189		3
G1K331;G1K329;P84229;C6ZL36;Q0	Histone H3 (Fragment)	LOC417953;H3-I;	0.883	0.871		0.328	-0.179	-0.199		-1.607	3
P50890;Q6SVA6;F1NKH1	40S ribomal protein SA	RPSA	0.885	1.044	1.328	0.961	-0.177	0.063	0.410	-0.058	4
E1BQB6;F1NC38;Q5ZJ93	Uncharacterized protein	ACADL;RCJMB04	0.885	0.896		0.119	-0.177	-0.158		-3.071	3
E1BR10	Uncharacterized protein	PRDX3	0.890	0.805	0.664	1.493	-0.168	-0.313	**-0.591**	0.578	4
B0LVF9;B0LVG0;Q9PRV4	Microtubule-associated protein	MAPT	0.893	1.029	0.374		-0.163	0.041	**-1.419**		3
Q5ZKM2;Q90835;F1N9H4;Q6PTX1	Elongation factor 1-alpha	RCJMB04_10b5;(	0.901	1.068	0.500	1.501	-0.151	0.095	**-1.000**	0.586	4
G1K332;F1NCK6;P62801;P70081;Q(	Histone H4 (Fragment)	H4;H4-I;H4-VIII	0.903	0.817	3.305	0.259	-0.147	-0.291	1.724	-1.949	4
F1P2T7;O93378;B9VVJ4;Q9PWP7;Q	Uncharacterized protein (Fragment)	STAT5B;Stat5	0.903	1.350		0.600	-0.147	0.433		-0.737	3
F1P4H4;Q5ZIM7	Uncharacterized protein	TXNDC5;RCJMB0	0.904	1.858	0.085	5.081	-0.146	**0.893**	**-3.558**	**2.345**	4
Q5ZLN9;F1ND94	Uncharacterized protein	RRAGC;RRAGD	0.905	1.196	3.936		-0.144	0.259	1.977		3
E1BTR4	Uncharacterized protein	SPINK4	0.905		3.869		-0.143		1.952		2
E1C8P2	Uncharacterized protein	PAPSS1	0.906		2.467	0.347	-0.143		1.303	-1.526	3
P00548	Pyruvate kinase muscle isozyme	PKM2	0.907	1.089	3.027	0.646	-0.141	0.123	1.598	-0.631	4
Q9IAY5	Protein syndesm	SDOS	0.915		1.262		-0.128		0.336		2
P18359	Destrin	DSTN	0.917	0.839	0.398		-0.125	-0.254	**-1.328**		3
F1N964	Uncharacterized protein (Fragment)	GSR	0.920		0.169	7.388	-0.120		**-2.562**	**2.885**	3
P00356;F1NH87	Glyceraldehyde-3-phphate dehydrogenase	GAPDH	0.922	0.897	1.376		-0.118	-0.156	0.460		3
E1BSQ4;E1BSQ3;E1C8Y4;E1C8Z0;F1	Dolichyl-diphphooligaccharide--protein glycyltransferase subunit 2	RPN2;RCJMB04_	0.926	1.045			-0.111	0.064			2
F1N887	Uncharacterized protein (Fragment)	KIAA0391	0.927	0.927	5.864	0.306	-0.110	-0.110	2.552	-1.708	4
E1C6N0	Uncharacterized protein	PSMD14	0.929	1.116			-0.106	0.159			2
Q8AWB4;Q5ZLZ9;F1NV29	Putative uncharacterized protein POFUT1	POFUT1;RCJMB0	0.930	0.714	4.585	0.166	-0.105	-0.486	2.197	-2.592	4
F1NHF1	Uncharacterized protein (Fragment)	ATP5J2	0.931	0.931			-0.103	-0.103			2
F1NX56;F1P4M6;Q5ZKF0	Uncharacterized protein	OGT	0.932	0.895	2.222	0.483	-0.102	-0.160	1.152	-1.051	4
P0C7A1;F1NLP8	Cytolic endo-beta-N-acetylglucaminidase	ENGASE	0.934		0.463	2.253	-0.099		**-1.112**	**1.172**	3
E1C525	Uncharacterized protein	EXOSC10	0.939		4.505	0.335	-0.092		2.172	-1.577	3
Q5ZKC9;F1NPX9;Q9PS14	14-3-3 protein zeta	YWHAZ	0.939	0.852			-0.091	-0.231			2
F1P2C8;F1P399;Q5ZKA2	Isoleucine--tRNA ligase, mitochondrial (Fragment)	IARS2	0.942	1.487	1.921	0.487	-0.087	0.572	0.942	-1.037	4
Q5ZIA5	Coatomer subunit beta	COPB1	0.942	0.992	0.923	0.579	-0.086	-0.012	-0.115	-0.790	4
E1BUD8	Uncharacterized protein	SEC24C	0.946	1.133			-0.081	0.181			2
F1NW43;F1P4U1	Pyruvate kinase (Fragment)	PKM2	0.947	1.095	2.987	0.597	-0.078	0.131	1.579	-0.745	4
Q9PTR5	Lissencephaly-1 homolog	PAFAH1B1	0.948		3.974		-0.077		1.991		2
Q5ZJK1;F1NCM4;F1NEI6;Q5ZIA6	THO complex subunit 5 homolog	THOC5;RCJMB04	0.953	1.313		0.314	-0.070	0.393		-1.670	3
F1NG30	Uncharacterized protein	NAA25	0.956	0.939			-0.064	-0.090			2
D0EKR3	Peptidyl-prolyl cis-trans isomerase	PPCTI	0.957	1.330	0.190	3.249	-0.063	0.411	**-2.394**	**1.700**	4
F1NDB3;P21265;Q683M9;E1BR00;C	Adenyluccinate lyase	ADSL	0.957	0.840			-0.063	-0.251			2
F1NSZ4	Uncharacterized protein (Fragment)	NPEPL1	0.958	1.345	0.864	2.804	-0.062	0.427	-0.210	**1.488**	4
Q5ZI56;E1C3K3;F1NYL7	Putative uncharacterized protein	RCJMB04_30d2;(	0.959	1.504			-0.060	**0.588**			2

F1NQZ9	Uncharacterized protein (Fragment)	GCLC	0.962		0.624		-0.055		**-0.680**		2
F1NT20;E1BV95;Q5ZHQ4		ACAT2	0.964	1.217		1.174	-0.052	0.283		0.231	3
P05094;P05094-2;P20111-2;P20111	Alpha-actinin-1	ACTN1	0.965	0.505		0.133	-0.051	-0.986		-2.909	3
F1NX13;Q5H7M6	Uncharacterized protein	DRG1;drg1	0.966	0.980	1.760	0.751	-0.049	-0.028	0.816	-0.413	4
Q5ZJK8;F1NK38;F1NZN1	T-complex protein 1 subunit eta	CCT7	0.968	0.855	2.681	1.837	-0.047	-0.225	1.423	0.877	4
Q6EE31;F1P1N9;F1NEF2	T-complex protein 1 subunit theta	CCT8	0.973	0.741		1.040	-0.040	-0.432		0.056	3
Q5ZHN0;F1NLB6;E1C059	Putative uncharacterized protein	RCJMB04_35e5;I	0.973	1.306			-0.039	0.385			2
F1NK96;F1N966;Q5F472	Uncharacterized protein	PDIA6	0.976	1.056	0.260	1.872	-0.035	0.078	**-1.941**	**0.905**	4
E1BY12;E1BXC1;F1NCI1;F1NCR9	Uncharacterized protein	AP2B1;AP1B1	0.977	1.063		1.396	-0.034	0.088		0.481	3
Q90734	Alpha-actinin-4	ACTN4	0.983	0.744	5.163	0.120	-0.024	-0.426	2.368	-3.064	4
F1NEG6;F1NJJ5;Q6WNG8;F1NJJ4;Q	Uncharacterized protein	HNRNPH1;RCJMI	0.988	0.736		0.731	-0.018	-0.443		-0.452	3
Q5ZLD5;F1NUL8;F1P4N6	Putative uncharacterized protein	RCJMB04_6k8;W	0.988	1.135			-0.018	0.183			2
Q8JFP1;F1NTS2;Q5ZM36;E1BSE5	Eukaryotic initiation factor 4A-II	EIF4A2	0.989	1.239		0.702	-0.016	0.310		-0.510	3
F1N8K4;Q5ZMS2	Platelet-activating factor acetylhydrolase IB subunit beta	PAFAH1B2	0.992	0.971	0.477	1.446	-0.011	-0.043	**-1.068**	0.532	4
G1K326;P0CB50	Peroxiredoxin-1 (Fragment)	PRDX1	0.994	0.812	1.278		-0.008	-0.300	0.354		3
P47826;F1NB66	60S acidic ribomal protein P0	RPLP0	0.995	1.257	2.427	0.650	-0.007	0.330	1.279	-0.620	4
F1NNP8	Uncharacterized protein (Fragment)	TSR1	0.996	1.030	0.578	0.606	-0.005	0.043	**-0.792**	-0.724	4
E1BV44	Uncharacterized protein	CSE1L	0.999	0.936	0.254	0.821	-0.002	-0.096	**-1.979**	-0.285	4
G8JL27;P63247	Guanine nucleotide-binding protein subunit beta-2-like 1 (Fragment	GNB2L1	1.003	0.990	4.166	0.560	0.004	-0.014	2.059	-0.837	4
F1NRW7;F1NWP3;O73885;B3VHV2	Heat shock cognate 71 kDa protein (Fragment)	HSPA8	1.004	1.151	1.201	1.913	0.005	0.203	0.264	**0.936**	4
E1BVP1;E1C8K7	Uncharacterized protein	EIF5B	1.004	0.775	4.431		0.005	-0.367	2.148		3
P11501;F1NVN4;Q5ZKZ3;Q2XQE5;F	Heat shock protein HSP 90-alpha	HSP90AA1	1.005	1.143			0.008	0.193			2
F1N9X5;Q5ZJC1;F1NPA9;Q6EE58	Uncharacterized protein	RPS3;RCJMB04_	1.006	1.272	2.091	0.720	0.009	0.347	1.064	-0.473	4
Q5ZLC5	ATP synthase subunit beta, mitochondrial	ATP5B	1.008	0.943	0.478	0.637	0.011	-0.085	**-1.065**	-0.650	4
Q6EE30	Eukaryotic translation elongation factor 1	eTEF1	1.011	0.871	1.105	0.915	0.015	-0.199	0.144	-0.128	4
Q8JG64	Protein disulfide-isomerase A3	PDIA3	1.014	1.196	0.326	0.964	0.019	0.258	**-1.617**	-0.053	4
Q5ZJQ5;Q5ZJQ6	Putative uncharacterized protein	RCJMB04_16g5;I	1.019		4.056	0.693	0.027		2.020	-0.529	3
F1NU17;P51903	Phphoglycerate kinase	PGK1;PGK	1.019	0.946	0.461	1.567	0.028	-0.080	**-1.119**	0.648	4
F1N941;E1C0S2;Q5ZK88	Paraspeckle component 1	PSPC1	1.022	0.728	1.686	0.794	0.031	-0.459	0.754	-0.333	4
F1P1A8	Uncharacterized protein (Fragment)	SMARCC1	1.024	1.232	0.698	1.220	0.034	0.301	-0.518	0.287	4
Q5ZKN9	Putative uncharacterized protein	RCJMB04_9n20	1.031	0.898			0.044	-0.155			2
Q5ZMC1;Q5ZMA1	Uncharacterized protein	DDX19B	1.035	1.177	2.276		0.050	0.235	1.187		3
A3R064	Docking protein 3	DOK3	1.039		2.201		0.055		1.138		2
E1C266	Uncharacterized protein	YARS2	1.043		4.327		0.061		2.113		2
Q5ZL72	60 kDa heat shock protein, mitochondrial	HSPD1	1.044	0.866		0.859	0.062	-0.208		-0.219	3
F1NIP5;E1BRQ0;Q5ZI49	Ribe-phphate pyrophphokinase (Fragment)	PRPS1	1.047	0.742		0.853	0.067	-0.431		-0.230	3
Q90WD0;F1NW48	Actin-related protein 3	ACTR3;ACTR3B	1.048	1.049			0.068	0.069			2
F1P555;Q91017	Uncharacterized protein	SFPQ	1.050	0.623	2.377	0.398	0.070	-0.682	1.249	-1.329	4
Q5F424	Uncharacterized protein	CCT2	1.050	1.087	2.613	0.546	0.070	0.121	1.385	-0.872	4
Q90XD9	Transcriptional coactivator p100 (Fragment)		1.061	0.984	1.796	0.652	0.085	-0.024	0.845	-0.617	4

Q5F3L2;F1NYE5;F1NDI3	Putative uncharacterized protein	RCJMB04_14f6;S	1.063	1.172	0.672	0.877	0.089	0.229	**-0.574**	-0.189	4
F1P582;D0VX29	Uncharacterized protein	UQCRC2	1.066	0.766	2.312	0.639	0.092	-0.384	1.209	-0.646	4
F1NIQ3;Q5F3V3	Sister chromatid cohesion protein PDS5 homolog A	PDS5A	1.069	1.112	0.955	0.619	0.096	0.153	-0.067	-0.692	4
F1NGA2;F1NI22;Q8UVX3;Q9DDH7;(	ATP synthase subunit alpha (Fragment)	ATP5A1	1.074	0.912	0.551	0.571	0.103	-0.133	**-0.860**	-0.810	4
Q5ZLF2;Q8JH64	Tyrosine-protein kinase BTK	BTK	1.074	0.844	5.331		0.103	-0.245	2.414		3
E1C6E1	Uncharacterized protein	PLCG2	1.076	0.832	8.140		0.105	-0.266	3.025		3
F1NJX8	Uncharacterized protein (Fragment)	FNTA	1.080	1.036	2.516	1.140	0.111	0.051	1.331	0.189	4
F1P2L1;Q5ZMF5;E1C2S5;E1C8M9	Uncharacterized protein	CANX;RCJMB04_	1.081	0.772	0.532	1.769	0.112	-0.374	**-0.911**	0.823	4
Q98UJ8	Branched-chain alpha-keto acid dehydrogenase E1-alpha subunit (Fragment)		1.081		2.673		0.112		1.419		2
F1NRD9;F1NV49;Q90WU3	ATP-dependent RNA helicase DDX1 (Fragment)	DDX1	1.082	0.742	2.220		0.113	-0.430	1.150		3
Q5ZI29;F1NFD9;F1NJ97	Putative uncharacterized protein	RCJMB04_31a13	1.096	0.762	0.994	0.856	0.132	-0.392	-0.009	-0.225	4
Q5ZLW7;F1NK51	Putative uncharacterized protein	RCJMB04_4j8;LU	1.106	1.121	1.954		0.146	0.165	0.966		3
E1C7D4;Q5ZIZ6;E1C7L0	Uncharacterized protein	CTBP1	1.108	0.843	1.339	0.676	0.147	-0.246	0.421	-0.565	4
Q5F3D2	Uncharacterized protein	HNRNPH3	1.109	0.768	1.589	0.881	0.149	-0.381	0.668	-0.183	4
F1NJL0	Uncharacterized protein (Fragment)	ATXN2	1.114	0.907	2.752	0.679	0.155	-0.141	1.461	-0.559	4
F1NM59;E1BQE4;E1BQE3;E1BQE2	Uncharacterized protein (Fragment)	RBM27	1.121	0.931			0.165	-0.103			2
F1P2P4	Uncharacterized protein (Fragment)	CARKD	1.122	1.081	1.103	1.157	0.166	0.113	0.142	0.210	4
P09531	Transforming growth factor beta-1 (Fragment)	TGFB1	1.128	1.538		2.400	0.173	**0.621**		**1.263**	3
O57660;F1NI29	CFR-associated protein p70	HADHA	1.128	1.044			0.174	0.062			2
P84175	40S ribomal protein S12	RPS12	1.129	1.493			0.175	**0.578**			2
Q5ZJQ2	Phenylalanine--tRNA ligase alpha subunit	FARSA	1.134	2.023	4.184		0.181	**1.016**	2.065		3
F1NU36;F1NDD6;O57378	Uncharacterized protein (Fragment)	LRPAP1;rap	1.137	1.152		1.861	0.185	0.204		**0.896**	3
F1N9I5;Q9PW24	Uncharacterized protein	ELAVL1;HuA	1.141	0.673	2.993		0.190	-0.571	1.582		3
F1P0Y4;F1P0Y5;Q5F447;F1NW30	Uncharacterized protein	SFRS15;RCJMB04	1.147	0.975	2.308	0.661	0.198	-0.036	1.206	-0.597	4
F1NBL6	Uncharacterized protein (Fragment)	XPO5	1.156	0.826	0.427	0.682	0.209	-0.275	**-1.227**	-0.552	4
Q5ZIZ5	Uncharacterized protein	NONO	1.157	0.725	4.094	0.306	0.211	-0.463	2.033	-1.707	4
F1NVF0;Q5ZL51	Uncharacterized protein	VPS35;RCJMB04	1.158	0.943	0.573	0.989	0.211	-0.084	**-0.804**	-0.016	4
Q5ZJ61	Uncharacterized protein	FARSB	1.160	0.792	3.939	0.434	0.214	-0.336	1.978	-1.205	4
Q5ZM62;F1NIH9;F1NII0;F1NHT0;Q5	Putative uncharacterized protein	RCJMB04_2p22;	1.160	0.843	4.012		0.214	-0.247	2.004		3
F1NP63	Uncharacterized protein (Fragment)	KPNB1	1.163	0.809	1.783	0.826	0.218	-0.306	0.834	-0.276	4
Q5ZHZ0	Spliceome RNA helicase DDX39B	DDX39B	1.180	0.710	0.533	0.717	0.239	-0.494	**-0.908**	-0.480	4
E1C7K8	Uncharacterized protein	NDUFAF4	1.181	0.842		0.765	0.240	-0.247		-0.386	3
Q98TY6;F1NXW5;F1NL77	Tyrosine kinase negative regulator Cbl	CBL;CBLB	1.184	0.731			0.244	-0.452			2
E1C4M0	Uncharacterized protein	RPS2	1.197		1.584	0.386	0.259		0.663	-1.373	3
Q5ZIV6;F1NSV7;F1NHF5;F1NHM6	Putative uncharacterized protein	RCJMB04_23e15	1.214	0.886	1.276	0.716	0.280	-0.174	0.352	-0.482	4
Q98SE2;Q98TR0	U2snRNP auxiliary factor small subunit	U2AF1	1.216	1.382			0.282	0.466			2
E1BSL7	Uncharacterized protein	ERP44	1.217	0.767	0.711	1.896	0.283	-0.383	-0.492	**0.923**	4
E1C2C3	Uncharacterized protein	SF3B1	1.226	0.687	4.310	0.720	0.293	-0.541	2.108	-0.474	4
F1P1A5	Uncharacterized protein (Fragment)	TKT	1.233	0.566	2.740	0.153	0.302	-0.822	1.454	-2.708	4
Q5ZLC7	Microtubule-associated protein RP/EB family member 1	MAPRE1	1.234	1.057			0.303	0.080			2

F1NZ86;Q5ZM98	Stress-70 protein, mitochondrial	HSPA9	1.255	0.983	1.008	1.435	0.328	-0.025	0.012	0.521	4
P00940	Triephphate isomerase	TPI1	1.260	0.698		1.693	0.333	-0.519		0.759	3
F1NIH4;F1NFY8;Q90687	Tyrosine-protein phphatase non-receptor type 11 (Fragment)	PTPN11	1.263	1.080	3.375		0.336	0.111	1.755		3
Q5ZLE8;F1NY80;E1C094	Putative uncharacterized protein	RCJMB04_6i5;DH	1.265		4.177		0.339		2.063		2
G1K342;F1NYB1;P09206;P09653;F1	Uncharacterized protein	TUBB2C	1.265	0.933	1.217	0.883	0.339	-0.100	0.283	-0.179	4
Q6XD56;F1NNR6	SWAN ribonucleoprotein	RBM12	1.265	0.886		1.159	0.339	-0.174		0.213	3
F1N8Z4;Q5ZIC4	Uncharacterized protein	RUVBL1;RCJMB0	1.272	0.623	2.327	0.360	0.347	-0.682	1.218	-1.474	4
Q5ZL82	Isocitrate dehydrogenase [NADP]	RCJMB04_7e11	1.291	0.937	0.837	0.694	0.368	-0.093	-0.256	-0.527	4
Q5F4B4	Cell-cycle related and expression-elevated protein in tumor	RPRD1B	1.303	0.546		0.222	0.382	-0.872		-2.174	3
E1C3A1	Uncharacterized protein	KIF5B	1.303	0.879	2.137	0.449	0.382	-0.186	1.096	-1.155	4
Q5ZKL0	Uncharacterized protein	MCM5	1.305	0.772	6.235		0.384	-0.374	2.640		3
Q5ZLZ1;F1NB20	Putative uncharacterized protein	RCJMB04_4e20;I	1.312	0.780			0.392	-0.359			2
F2Z4M0;Q9W7I5;Q9I8G9	Histone-binding protein RBBP4	RBBP4;RBBP7	1.319	1.123	3.467	0.664	0.400	0.167	1.794	-0.591	4
F1NHL2	ubiquitin ligase complex	CAND1	1.322	0.852	0.673	0.875	0.403	-0.230	**-0.572**	-0.193	4
P79994;F1NPT1;F1NXP4;Q6T722	DNA topoisomerase I	TOP1	1.329	0.815	2.210	0.117	0.410	-0.296	1.144	-3.089	4
Q5F491;F1NIX1;F1NIX2	Putative uncharacterized protein	RCJMB04_2a4;D	1.330	0.884			0.412	-0.177			2
Q5ZLQ7;F1NNL9	Putative uncharacterized protein	RCJMB04_5d7;G	1.332	1.180	0.665	1.047	0.413	0.239	**-0.589**	0.067	4
F1NFJ0;Q5ZMN2	DNA replication licensing factor MCM3	MCM3	1.370	0.741	4.389	0.728	0.454	-0.433	2.134	-0.457	4
F1ND23	Uncharacterized protein (Fragment)	NDUFS3	1.370	0.875	2.970		0.454	-0.193	1.570		3
F1NKB6;E1BXW3;F1NW16;Q8AYP9	6-phphofructokinase	PFKL;pfk	1.371		3.839		0.455		1.941		2
Q5ZK92;F1NCJ3;E1C6S3;Q5ZK92-2	Spastin	SPAST	1.371	0.846		1.994	0.455	-0.241		**0.996**	3
F1NEE3;Q5ZM37;F1NLX5	Uncharacterized protein	PCID2;RCJMB04_	1.371	1.357	2.573		0.456	0.440	1.363		3
Q5ZL34;F1NGU9	Cleavage and polyadenylation specificity factor subunit 6	CPSF6	1.388	0.891	0.735	1.048	0.472	-0.167	-0.444	0.067	4
F1NCI5	Uncharacterized protein (Fragment)	HNRNPA0	1.422	0.547	3.758	1.071	0.507	-0.871	1.910	0.099	4
Q5F426	Uncharacterized protein	PDHA1	1.431	0.632	0.568	1.646	0.517	-0.662	**-0.815**	0.719	4
F1NJ40;Q5F485;F1P0H1	ATP-dependent RNA helicase DDX42	DDX42	1.466	1.364		0.592	0.552	0.448		-0.757	3
Q5ZMT0;F1NGW3;F1P2P9	14-3-3 protein epsilon	YWHAE	1.468	0.700			0.553	-0.515			2
P09244	Tubulin beta-7 chain	TUBB7	1.474	0.631			0.559	-0.665			2
F1NKR1	Uncharacterized protein (Fragment)	MAN2A1	1.481	0.730	0.614	1.498	0.566	-0.454	**-0.703**	0.583	4
E1C538	Uncharacterized protein	NUDT21	1.482	0.966	1.753	0.877	0.568	-0.050	0.809	-0.190	4
E1C2U4	Uncharacterized protein	LOC421110	1.487	0.781	3.985	0.445	0.572	-0.356	1.994	-1.168	4
Q5ZMM8	Uncharacterized protein	LOC416354	1.508	1.253	3.102		0.592	0.325	1.633		3
F1NXK0;Q5ZJV4	Uncharacterized protein	C10orf119;MCM	1.518	0.994	3.848	0.661	0.602	-0.008	1.944	-0.597	4
E1C4U7	Uncharacterized protein	NDUFB3	1.523	0.818			0.607	-0.289			2
P84172	Elongation factor Tu, mitochondrial (Fragment)	TUFM	1.536	1.209	4.483	0.950	0.620	0.274	2.165	-0.074	4
O13016;E1BWI7	Tyrosine-protein phphatase non-receptor type 1	PTPN1	1.548		2.469		0.630		1.304		2
F1P4A7;F1NQU7;Q5KTT9	Eukaryotic translation initiation factor 4 gamma 2 (Fragment)	EIF4G2;NAT1	1.560	0.967			0.641	-0.049			2
Q5ZL58	Uncharacterized protein	SNRPD3	1.563		2.797		0.644		1.484		2
F1NDS2	Uncharacterized protein (Fragment)	MRPL39	1.617		5.821	0.143	0.693		2.541	-2.808	3
F1NXA5;Q5ZI60;Q5ZM02;Q5ZLS1;F1	Uncharacterized protein (Fragment)	CORO1C	1.649	0.380	3.285	0.179	0.721	-1.396	1.716	-2.485	4

F1NJG2;Q8AYC6	Uracil-DNA glycylase	UNG;ung	1.650	0.328	5.257	0.134	0.722	-1.609	2.394	-2.895	4
E1BYM1	mRNA cap guanine-N7 methyltransferase	RNMT	1.675	0.721	3.276	0.174	0.744	-0.472	1.712	-2.520	4
Q6U7I1;F1N909;F1P4U4;Q6U7I1-2;	Ubiquitin carboxyl-terminal hydrolase 7	USP7	1.690	0.615			0.757	-0.702			2
F1N945;F1NE00	Uncharacterized protein (Fragment)	KIF5C	1.730	0.651	3.919	0.265	0.790	-0.620	1.970	-1.914	4
F1NWF6;Q5ZJU3	Asparagine synthetase [glutamine-hydrolyzing]	ASNS	1.846	0.891	2.691	0.432	0.884	-0.166	1.428	-1.209	4
P00340;E1BTT8	L-lactate dehydrogenase A chain	LDHA	1.918	0.539	5.168	0.368	0.940	-0.891	2.370	-1.442	4
E1C3R4;E1C6R3	Uncharacterized protein	LANCL2	2.090		3.593	0.389	1.064		1.845	-1.361	3
E1C8Q3	Uncharacterized protein	ALDH18A1	2.195	1.100	4.346	1.087	1.134	0.137	2.120	0.121	4
Q5F3C0;E1BVJ5	Putative uncharacterized protein	RCJMB04_22h1;	2.229	0.411	4.028	0.322	1.156	-1.284	2.010	-1.633	4
Q9PW38;F1P187;E1C756	Gephyrin	GPHN	2.522	0.594	0.591		1.335	-0.751	-0.759		3
E1BXF5;E1C6D1	Microtubule-associated protein	MAP2	2.663	0.213	3.106	0.564	1.413	-2.233	1.635	-0.825	4
Q5ZKK1;F1NRY2;F1N8Q2	Microtubule-associated protein RP/EB family member 2	MAPRE2	2.805	0.385	2.869		1.488	-1.376	1.520		3
Q5ZLV5;F1P472;F1P463	Uncharacterized protein	RCJMB04_4l9;CNDP2			0.161	3.389			-2.639	1.761	2
Q5ZLN1;F1NHM9;F1NQ41	Phosphoglycerate mutase 1	PGAM1			0.235	10.508			-2.089	3.393	2
F1NDI7;F1NC33;Q04619	Heat shock cognate protein HSP 90-beta	HSP90AB1		0.860	0.318	0.662		-0.217	-1.652	-0.595	3
Q5ZM75	Putative uncharacterized protein	RCJMB04_2o24			0.342	1.496			-1.548	0.581	2
E1C6N0;Q5ZJ35	Uncharacterized protein	PSMD14			0.350	1.702			-1.513	0.767	2
E1BZJ3	Uncharacterized protein	NPLOC4			0.355	1.327			-1.493	0.408	2
P13648	Lamin-A	LMNA			0.358	7.185			-1.481	2.845	2
E1C3A9;F1NNX1;P81021	Vigilin	HDLBP			0.361	0.616			-1.468	-0.700	2
Q5ZJV5	Uncharacterized protein	COX4I1			0.380	1.359			-1.398	0.443	2
P23007	Citrate synthase, mitochondrial	CS			0.407	19.213			-1.296	4.264	2
F1NU73;Q5ZMF6	Uncharacterized protein	TRAP1;RCJMB04_2d7		0.790	0.443	0.657		-0.341	-1.174	-0.606	3
E1C516	Uncharacterized protein	PROSC		1.013	0.456	1.672		0.019	-1.134	0.741	3
F1NV05;F1NIJ6;Q5ZMU3;CON__Q3	Glucose-6-phosphate isomerase	GPI;RCJMB04_1c14		0.851	0.459			-0.232	-1.124		2
P09102;F1N9H3;P12244	Protein disulfide-isomerase	P4HB			0.479	3.023			-1.063	1.596	2
F1P5J5;Q5ZKF3	Uncharacterized protein	AARS;RCJMB04_11d4		1.176	0.505	0.452		0.234	-0.984	-1.147	3
Q9YGW6;F1P3H9	Ezrin	EZR			0.526	0.864			-0.927	-0.211	2
P54939;E1BWD2;E1C2S1;E1BUK3	Talin-1	TLN1		0.416	0.531	2.243		-1.265	-0.914	1.165	3
F1NQP8;E1BRG8;Q5F443	Uncharacterized protein	CCDC6;RCJMB04_3i14		1.712	0.573	3.605		0.775	-0.803	1.850	3
F1NRK5;Q5ZHR4;E1C7P8;E1C1B7;E1	Uncharacterized protein	GARS;RCJMB04_34b10		0.834	0.674	0.807		-0.262	-0.570	-0.310	3
Q5ZK08;F1NBD1	Putative uncharacterized protein	RCJMB04_13p14;NARS			0.730	1.298			-0.453	0.376	2
F1NER5	Uncharacterized protein	CHID1		0.784	0.759	1.086		-0.351	-0.399	0.119	3
F1P4U8	Uncharacterized protein				0.902	1.370			-0.149	0.454	2
Q5ZMW1;Q8AYI3	Aconitase	ACO2			0.904	1.695			-0.146	0.761	2
F1NXD7	Uncharacterized protein	SEC23IP			0.994	1.375			-0.009	0.459	2
E1C555;F1NJN5	Uncharacterized protein	SMARCD2;SMARCD3		1.099	1.140	0.671		0.136	0.189	-0.576	3
F1NNI2	Uncharacterized protein	LONP1			1.191	0.364			0.252	-1.459	2
E1BVN5	Uncharacterized protein	PLRG1			1.200	0.322			0.263	-1.637	2
Q5ZLH1	Uncharacterized protein	RPA2		1.259	1.217	1.238		0.333	0.283	0.308	3

F1NQH4;Q90YB6;Q90WG3;F1NCE3	Formin binding protein 11-related protein	PRPF40A;FBP			1.293	1.170			0.371	0.227	2
Q9YHT1;F1NPJ4;F1NHM1	Succinate dehydrogenase [ubiquinone] flavoprotein subunit, mitoch	SDHA		1.834	1.440			**0.875**	0.526		2
F1NZ25	Uncharacterized protein	EIF3B			1.446	0.439			0.532	-1.187	2
F1NRB9	Tubulin alpha 3	TUBA3E		0.855	1.507	0.617		-0.226	0.591	-0.697	3
F1NF75	Uncharacterized protein	EEPD1		1.185	1.559	0.380		0.245	0.640	-1.396	3
Q5ZKP8;F1NE72;E1C3E0;Q8JGU2;RI	Lysyl-tRNA synthetase	RCJMB04_9m1;KARS		0.913	1.579	0.751		-0.131	0.659	-0.413	3
P10360	Cellular tumor antigen p53	TP53		0.443	1.627	0.186		-1.175	0.702	-2.429	3
E1C3Y5	Protein transport protein Sec23A	SEC23A		1.605	1.669	0.114		**0.683**	0.739	-3.134	3
E1BTG1	Uncharacterized protein	RPL12			1.688	3.246			0.755	**1.699**	2
E1BVP9;F1P2M7;Q5F399	Uncharacterized protein	PRPF38B			1.704	0.831			0.769	-0.267	2
Q5F387	Uncharacterized protein	SF3A3			1.737	0.837			0.796	-0.257	2
F1N8J0;Q5ZLW8	Uncharacterized protein	ACAA2;RCJMB04_4j7		1.141	1.742			0.191	0.801		2
F1NCA1;Q5ZL28	Uncharacterized protein	CCNK;RCJMB04_8b8		0.933	1.837			-0.101	0.878		2
E1BRA6	Uncharacterized protein	DNAJC10		1.766	1.841	2.047		**0.820**	0.881	**1.033**	3
Q5F4A4;E1C707;Q5ZLI1;F1N808	Inosine-5-monophosphate dehydrogenase	IMPDH2		0.303	1.885	0.498		-1.724	0.914	-1.006	3
Q5ZKF7;E1C6M5;F1NIK2	Putative uncharacterized protein	RCJMB04_11c15;CBL		0.857	2.079	0.790		-0.223	1.056	-0.340	3
E5DEA6;E5DFI5;E5DFL1;P18944;Q4	Cytochrome c oxidase subunit 2	COX2;MT-CO2;COII		1.183	2.081			0.242	1.057		2
E1BUH7	Uncharacterized protein	TMEM43			2.082	0.582			1.058	-0.781	2
F1NAC6;D0VX31	Uncharacterized protein	UQCRC1			2.117	0.167			1.082	-2.583	2
E1BY94	Uncharacterized protein	DEPTOR			2.132	0.330			1.092	-1.599	2
E1BQK9;F1P2U1	Uncharacterized protein	CUL4B;LOC418744		1.092	2.334	0.533		0.127	1.223	-0.908	3
Q5ZM11;F1NFP5	Arginine--tRNA ligase, cytoplasmic	RARS		0.830	2.476	0.403		-0.268	1.308	-1.312	3
E1BQE4;E1BQE3;E1BQE2	Uncharacterized protein	RBM26			2.563	1.211			1.358	0.276	2
F1P179;Q5ZJ86	Uncharacterized protein	EPRS;RCJMB04_20b9			2.645	0.296			1.403	-1.756	2
F1NLG5;F1NR54	Uncharacterized protein	SFXN1			2.761	0.290			1.465	-1.785	2
Q5ZLW6;F1P394	DNA ligase	RCJMB04_4j11;LIG3			2.826	0.245			1.499	-2.027	2
E1BQV7	Uncharacterized protein	SLK			2.938	0.253			1.555	-1.985	2
P18660	60S acidic ribosomal protein P1	RPLP1		0.787	2.959			-0.345	1.565		2
F1P257	Uncharacterized protein	TPP2		0.842	3.060	0.433		-0.247	1.614	-1.206	3
E1C262;E1BWK3	Uncharacterized protein	AP3D1			3.160	0.577			1.660	-0.794	2
E1BZ78	Uncharacterized protein	SMG8		0.835	3.207			-0.261	1.681		2
F1N8F0;Q9YGM2	Delta-9 desaturase	SCD		1.951	3.587	0.841		**0.964**	1.843	-0.250	3
E1C0J3;E1C0J4	Uncharacterized protein	UPF1			3.598	0.718			1.847	-0.477	2
Q5ZLR5	Cytochrome b-c1 complex subunit Rieske, mitochondrial	UQCRFS1		0.784	3.641			-0.351	1.864		2
E1BZ33	Uncharacterized protein	MYBBP1A			3.730	0.463			1.899	-1.109	2
E1C2I9	Uncharacterized protein	LARS			3.917	0.250			1.970	-2.000	2
E1BXI7	Uncharacterized protein	LARS2			3.920	0.477			1.971	-1.067	2
Q8UWC5	Nuclear protein matrin 3	MATR3		0.802	3.978	1.120		-0.318	1.992	0.163	3
Q5F3L1	Ribosomal protein S6 kinase alpha-5	RPS6KA5		0.772	4.016			-0.373	2.006		2
E1BQH2	Uncharacterized protein	IARS			4.444	0.312			2.152	-1.679	2

E1BZT5	Uncharacterized protein	PRPF8			4.535	0.485			2.181	-1.045	2
Q5ZHW3;E1C9E5	Uncharacterized protein	BUB3		0.740	4.773			-0.435	2.255		2
G1K2Y5;Q5F3X4	116 kDa U5 small nuclear ribonucleoprotein component	EFTUD2		1.380	4.973	0.717		0.464	2.314	-0.481	3
F1NBM0	Uncharacterized protein	OGFOD1		0.908	5.133			-0.139	2.360		2
F1N9U0	Uncharacterized protein	PRPF6			5.749	0.721			2.523	-0.473	2
F1P0W4	Uncharacterized protein	H1FX			5.777	0.250			2.530	-1.998	2
Q5ZK03	Protein transport protein Sec23A	SEC23A		0.967	6.214	0.245		-0.049	2.636	-2.028	3
A1KXL9;A5HUJ1;F1NBH9	XCTK2-like motor protein	KIFC1			8.377	0.164			3.066	-2.611	2
F1NVY9	Uncharacterized protein	ATPIF1		1.447		2.040		**0.533**		**1.028**	2
F1NJU7;Q5ZJ08;REV__A3FM20;REV	Tyrosine--tRNA ligase, cytoplasmic	YARS		1.443		2.133		**0.529**		**1.093**	2
E1BW50;Q5ZHU8;F1NRC7;F1NQD1;	Uncharacterized protein	CTNNBL1		1.318		1.464		0.398		0.550	2
O42395;O57348;F1N8C2;F1NI84;Q9	Cellular nucleic acid-binding protein	CNBP		1.288		1.746		0.365		0.804	2
P68034;P68139;P08023;P63270;F1I	Actin, alpha cardiac muscle 1;Actin, alpha skeletal muscle;Actin, aort	ACTC1;ACTA1;ACTA2;ACT		1.248		0.435		0.319		-1.201	2
Q5ZK40	SWI/SNF-related matrix-associated actin-dependent regulator of chr	SMARCB1		0.978		0.311		-0.032		-1.686	2
Q5ZKB2;F1NAG0;Q5ZKR8;REV__O4	Microtubule-associated protein	RCJMB04_11p11;MCM6		0.588		0.327		-0.767		-1.615	2
		median	0.962	0.997	1.841	0.730	-0.051	0.006	0.914	-0.453	
		1 sd	0.094	0.219	1.527	0.501	0.098	0.356	1.071	1.059	
		median +/- 1 sd	-0.001	1.089	-0.560	0.993	-0.001	0.123	-0.295	-2.157	

Key: **Bold** indicates ratio +/- 1sd of median. Heatmap annotation identifies enriched high confidence ratios (green) decreasing to low confidence or contradictory ratios (red). Green proteins are those also found in FLAG pulldowns
